# The HIV-1 Vpu Protein Induces Apoptosis in *Drosophila via* Activation of JNK Signaling

**DOI:** 10.1371/journal.pone.0034310

**Published:** 2012-03-29

**Authors:** Christelle Marchal, Gérald Vinatier, Matthieu Sanial, Anne Plessis, Anne-Marie Pret, Bernadette Limbourg-Bouchon, Laurent Théodore, Sophie Netter

**Affiliations:** 1 Laboratoire de Génétique et Biologie Cellulaire, EA4589, Université de Versailles St-Quentin-en-Yvelines, Versailles, France; 2 UFR des sciences, Université de Versailles St-Quentin-en-Yvelines, Versailles, France; 3 Institut Jacques Monod, CNRS, UMR 7592, Université Paris Diderot, Sorbonne Paris Cité, Paris, France; University of Massachusetts Medical School, United States of America

## Abstract

The genome of the human immunodeficiency virus type 1 (HIV-1) encodes the canonical retroviral proteins, as well as additional accessory proteins that enhance the expression of viral genes, the infectivity of the virus and the production of virions. The accessory Viral Protein U (Vpu), in particular, enhances viral particle production, while also promoting apoptosis of HIV-infected human T lymphocytes. Some Vpu effects rely on its interaction with the ubiquitin–proteasome protein degradation system, but the mechanisms responsible for its pro-apoptotic effects *in vivo* are complex and remain largely to be elucidated.

We took advantage of the *Drosophila* model to study the effects of Vpu activity *in vivo*. Expression of Vpu in the developing *Drosophila* wing provoked tissue loss due to caspase-dependent apoptosis. Moreover, Vpu induced expression of the pro-apoptotic gene *reaper*, known to down-regulate Inhibitor of Apoptosis Proteins (IAPs) which are caspase-antagonizing E3 ubiquitin ligases. Indeed, Vpu also reduced accumulation of *Drosophila* IAP1 (DIAP1). Though our results demonstrate a physical interaction between Vpu and the proteasome-addressing SLIMB/β-TrCP protein, as in mammals, both SLIMB/βTrCP-dependent and -independent Vpu effects were observed in the *Drosophila* wing. Lastly, the pro-apoptotic effect of Vpu in this tissue was abrogated upon inactivation of the c-Jun N-terminal Kinase (JNK) pathway. Our results in the fly thus provide the first functional evidence linking Vpu pro-apoptotic effects to activation of the conserved JNK pathway.

## Introduction

Viral invasion involves the expression of foreign genes that alter and constrain the host cellular machinery to propagate the life cycle of the virus. Studies in cell culture systems have shown that viral proteins develop complex interactions with cellular proteins thereby interfering with diverse cellular functions depending on the cell type or on the condition, acute or chronic, of the infection [1].

Human immunodeficiency virus type 1 (HIV-1) expresses a unique set of accessory proteins (Vif, Nef, Vpr and Vpu) that interfere with various host cell functions thereby optimizing replicative efficiency and viral pathogenesis. The 81-amino acid long viral type I membrane phosphoprotein U (Vpu) plays important roles in HIV-1 spreading and pathogenesis [2]. In particular, Vpu contributes to HIV-1-induced CD4 receptor downregulation and enhances virion release from infected cells [3]–[6]. A number of reports have shown the high complexity of the relationships between Vpu and cellular proteins of the host. They have highlighted the interaction between Vpu and the ubiquitylation/proteasome protein degradation system [2], [5]. Indeed, Vpu mediates retention and degradation of newly synthesized CD4 cellular receptor in the endoplasmic reticulum (ER) by promoting CD4 polyubiquitylation in the ER [7]–[9]. Cell-culture and *in vitro* experiments have demonstrated that Vpu can simultaneously bind CD4 and the β-Transducine repeat Containing Protein (β-TrCP), a F-box/WD40 substrate adaptor of the SCF (Skp-Cullin-F-box)/CRL1 (Cullin1-RING ubiquitin ligase) E3 ubiquitin ligase complex leading to CD4 ubiquitylation and subsequent proteasomal degradation [10]. The Vpu/β-TrCP interaction requires prior phosphorylation of Vpu by the casein-kinase II at a pair of serine residues (Ser52 and Ser56) within the cytoplasmic domain of Vpu. In cells arrested in early mitosis, the phosphorylation of another serine (Ser61) in Vpu may trigger its proteasomal degradation through an unknown E3-ubiquitin ligase, distinct from the SCF/CRL1-β-TrCP complex [11].

Recruitment of β-TrCP was also found to be required for Vpu-mediated BST2/Tetherin degradation [12]–[14]. BST2/Tetherin is a cellular factor responsible for inhibition of HIV-1 particle release, and its function is counteracted by that of Vpu [15], [16]. Vpu-induced BST2/Tetherin degradation did not entirely account for the anti-BST2/Tetherin activity of Vpu (for review, see [17]). This is further supported by results showing that β-TrCP is dispensable for Vpu to counteract the BST-2/Tetherin virion release block [18]. It has been suggested that other Vpu effects are also partly independent of its interaction with β-TrCP. For instance, Vpu was shown to bind to TASK1 (a cellular acid-sensitive K^+^ channel) which leads to formation of TASK1/Vpu hetero-oligomers that lack ion channel activity, thereby limiting TASK1 function through protein-protein interactions [19].

The regulation of HIV-1-induced apoptosis appears to be complex and Vpu might have multiple and opposite roles in this process. Vpu has been shown to contribute potently to the induction of apoptosis in HIV-infected T cells and in Hela-derived epithelial cells inducible for Vpu expression (Hela-CD4U) in a caspase-dependent manner [20], [21]. Sequestration of β-TrCP by Vpu inhibits β-TrCP, thus promoting the stabilization of certain of β-TrCP substrates such as I-κBα in cultured cells [21], [22]. By acting as a competitive inhibitor of β-TrCP, Vpu was shown to inhibit I-κBα degradation in HIV-1-infected cultured T cells or HeLa-CD4U cells, which resulted in a strong reduction in both TNFα- and HIV-induced activation of NF-κB activity [21]. Another study has shown that, by inhibiting the NF-κB-dependent expression of anti-apoptotic factors of the Bcl-2 family (Bcl-xL and A1/Bfl-1) and TNFR complex proteins (TNF receptor-associated factor 1, TRAF1), Vpu induced apoptosis through activation of the caspase pathway [20]. Likewise, very recently, Vpu was shown to compete for the interaction of tumor suppressor p53 with β-TrCP, leading to inhibition of p53 ubiquitylation and proteasomal degradation [23]. Consequent stabilization of p53 was shown to enhance p53-mediated apoptosis during HIV-1 infection. Vpu may also be able to induce apoptosis *via* other pathways since it was shown to render HIV-infected cells more susceptible to FAS-induced cell death [24].

“Viralized” transgenic *Drosophila* models have proven to be useful to study the function of various viral proteins at the level of a whole organism [25]–[27]. Three HIV viral proteins, Tat, Nef, and Vpu have already been studied using the *Drosophila* model. Expression of the Tat protein during fly oogenesis affected oocyte polarization resulting from interaction of Tat with tubulin [28] and in inhibition of ribosomal rRNA precursor processing in nurse cell nucleoli [29]. *Nef* expression induced caspase-dependent apoptosis in *Drosophila* developing wing cells *via* the activation of the c-Jun N-terminal Kinase (JNK) pathway and inhibited the *Drosophila* innate immune responses mediated by the Relish/NF-κB pathway [30]. Using transgenic flies expressing Vpu, we previously demonstrated that Vpu can also inhibit the *Drosophila* NF-κB-dependent immune response *in vivo* [31].

In the present study we show that Vpu expression in the fly disturbs normal development in particular reducing the size of the tissue where it is expressed, such as wing and eye. We also show that the interaction between Vpu and human β-TrCP [10] is conserved between Vpu and SLIMB, the *Drosophila* β-TrCP homolog, but this interaction is only partially responsible for the phenotypes induced by Vpu. Thus, the *Drosophila* model can be used for analysis of Vpu activity at the level of a whole organ, and for identification of novel functional interactions *in vivo*. We therefore carried out a genetic screen to identify modifiers of the Vpu-induced phenotypes and found that overexpression of *thread* encoding *Drosophila* Inhibitor of Apoptosis Protein 1 (DIAP1) very efficiently suppressed the wing phenotypes. Next, we demonstrated that Vpu expression in the developing *Drosophila* wing induced apoptosis cell-autonomously, which is also counteracted by *thread*/*diap1* overexpression. We further showed that Vpu activated expression of the pro-apoptotic *reaper* (*rpr*) gene and downregulated DIAP1 accumulation in this tissue. Finally, the activity of the JNK pathway was found to be necessary for Vpu-triggered apoptosis in the wing. Altogether the data reported here provide the first evidence of a functional link between Vpu-induced apoptosis and the activation of the conserved JNK signaling pathway.

## Results

### I - Vpu expression disrupts *Drosophila* development

**Figure 1:**
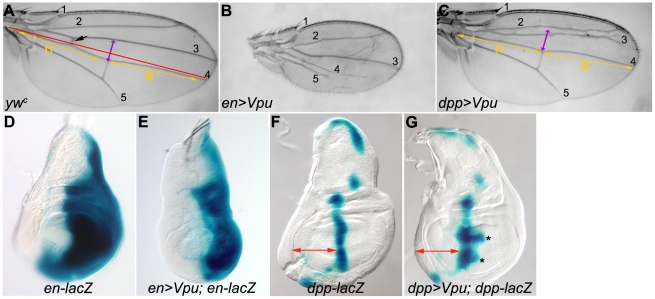
Vpu induces wing phenotypes in *Drosophila*. (A–C) Wings of *yw^c^* (A), *enGal4/+; UAS-Vpu/+* (*en>Vpu*, B) and *dpp-Gal4 UAS-Vpu/+* (*dpp>Vpu*, C) flies. Longitudinal veins are numbered. Black arrow in A: proximal cross-vein. Red line in A: boundary between the anterior and posterior compartments of the wing. Here and in all the other figures, wings are oriented with anterior up and proximal to the left and are at the same scale in a given figure. The length of the *dpp>Vpu* wing is traced by two segments l1 and l2, yellow arrows in C, placed on either side of the distal cross-vein. These arrows were placed in the corresponding positions in the image of the wild type wing in A for comparison. Both segments are shorter in the *dpp>Vpu* wing than in the wild type wing. The distance between veins 3 and 4 in the wild type wing was indicated with a purple arrow (A) and placed at the corresponding position in the *dpp>Vpu* wing (C) for comparison. Flies were raised at 26°C. (D–G) X-gal staining of wing imaginal discs expressing e*n-lacZ* or *dpp-lacZ* alone (D and F, respectively) or with *Vpu* in *en-Gal4/en-lacZ; UAS-Vpu/+* (E) and *dpp-lacZ*/+; *dpp-Gal4 UAS-Vpu*/+ flies (G). Asterisks in G: enlarged areas of the *dpp* expression domain. Imaginal discs (D–G) are at the same magnification. Here, and in all the other figures, wing imaginal discs are oriented with anterior left and dorsal up. The approximate width of the anterior compartment of the wing pouch in the *dpp>Vpu; dpp-lacZ* imaginal disc (G) is indicated with a red arrow which is placed at the equivalent position of a wild type disc (F) for comparison.

We expressed a transgene encoding Vpu [31] in various *Drosophila* tissues using the *Gal4/UAS* binary system [32]. Ubiquitous expression of Vpu led to lethality at the first instar larval stage, thereby indicating that Vpu interferes with essential developmental pathways. In order to address more precisely which cellular functions were affected, we restricted Vpu expression to specific territories in the developing larval wing primordium (wing imaginal disc) using *engrailed (en)*-*Gal4* and *decapentaplegic (dpp)*-*Gal4* transgenes which express Gal4 in the posterior compartment (shown in Figure 1D) and in a stripe of anterior compartment cells abutting the anteroposterior (A/P) compartment boundary (shown in Figure 1F) of the wing disc, respectively. In both cases, Vpu expression induced defects in the adult wing reflecting tissue loss and alteration of patterning during development (Figure 1). The expressivity of Vpu-induced phenotypes increased with the temperature (from very weak at 18°C, medium at 24.5°C, to very strong at 29°C; see next section and data not shown), indicating that they depend on Gal4 activity, which also increases with the temperature. Expression of Vpu with the *en*-*Gal4* driver led to a reduction of the entire wing along with additional tissue loss and vein defects in the posterior compartment (Figure 1A,B). Under the same conditions, the size of the posterior compartment of the larval wing imaginal disc (marked by the expression of an *en-lacZ* reporter) was reduced when compared to the wild type (Figure 1E,D). Expression of Vpu with *dpp-Gal4* also led to loss of wing tissue, mainly in the anterior region, between longitudinal vein 2 (L2) and L3, including part of L3, as well as loss of the proximal cross-vein between veins L3 and L4 associated with tissue loss between L3–L4 (Figure 1A,C black and purple arrow, respectively). Consistent with this adult wing phenotype, a slight reduction of the anterior part of the wing pouch was also observed in the corresponding wing imaginal discs (Figure 1F,G). However, in these same discs, the stripe of *dpp* expression (marked by the expression of a *dpp-lacZ* enhancer trap) appeared widened, in particular in two areas of the wing pouch (Figure 1G, asterisks). Developmental defects were also visible in the adult eye (*i. e.*, reduced, disorganized eyes resulting in a rough eye phenotype) using the *GMR-Gal4* driver (data not shown).

The expression of the viral protein Vpu during *Drosophila* development thus induced phenotypic defects in different cell types. In wing and eye, Vpu expression leads to a reduction in the size of the organ in which it was expressed, suggesting that it either induced cell death or reduced growth and cell proliferation.

### II - Vpu interacts with SLIMB/β-TrCP in *Drosophila* but Vpu-induced wing phenotypes are not fully dependent on this interaction

**Figure 2:**
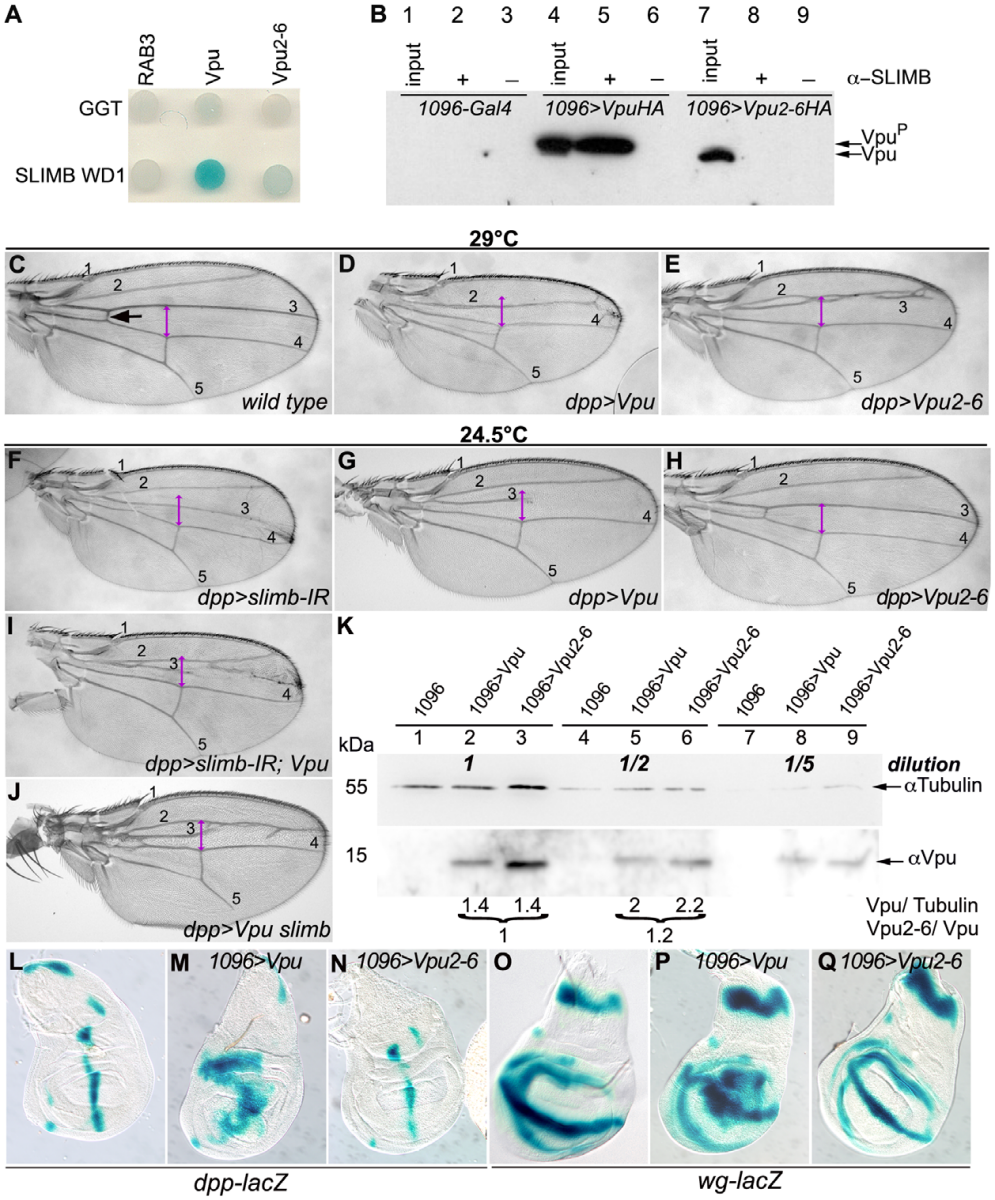
The conserved Vpu-SLIMB/β-TrCP interaction does not fully account for Vpu-induced phenotypes in *Drosophila*. (A) Two-hybrid assay. Yeast cells co-expressing the first WD domain of SLIMB (SLIMB WD1; row 2) or the human Geranyl-Geranyl TransferaseIIβ (GGT, row 1) along with the cytoplasmic domain of Vpu (column 2), Vpu2-6 (column 3) or human RAB3 (column 1). The blue color in the cells co-expressing Vpu and SLIMB WD1 developed too fast for the positive control (GGT/RAB3) to turn blue in the same amount of time, suggesting that the Vpu/SLIMB WD1 interaction is very strong. (B) Co-immunoprecipitation assay. Extracts from *MS1096-Gal4/+* (Lanes 1–3), *MS1096-Gal4/+; UAS-Vpu-HA/+* (Lanes 4–6) and *MS1096-Gal4/+*; *UAS-Vpu2-6-HA/+* (Lanes 7–9) larvae were immunoprecipitated with an anti-SLIMB antibody before immunoblotting with an anti-HA antibody. The difference in migration between Vpu and Vpu2-6 results from distinct phosphorylation levels (Vpu *vs.* Vpu^P^) on Ser52 and Ser56 [31]. *MS1096-Gal4* drives expression in the dorsal part of the wing pouch. (C–J) Wings of *yw^c^* (C), *dpp-Gal4 UAS-Vpu/+* (D,G), *UAS*-*Vpu2-6/+; dpp-Gal4/+* (E,H), *UAS-slimb-IR* (VDRC 107825)*/+; dpp-Gal4/+* (F), *UAS-slimb-IR* (VDRC 107825)*/+; dpp-Gal4 UAS-Vpu/+* (I) and *dpp-Gal4 UAS-Vpu/UAS-slimb* (J) flies. For comparison, phenotypes are shown at two temperatures (24.5°C or 29°C). Black and purple arrows: as in Figure 1A. (K) Quantification assay. Extracts from *1096-Gal4/+* (Lanes 1, 4, 7), *1096-Gal4/+; UAS Vpu/+* (Lanes 2, 5, 8) and *1096-Gal4/UAS-Vpu2-6* (Lanes 3, 6, 9) undiluted (Lanes 1–3), diluted 1/2 (Lanes 4–6) and 1/5 (Lanes 7–9) were immunoblotted with anti-Tubulin antibody (Loading control; top panel) and anti-Vpu antibody (Bottom panel). For quantification, concentrations of Vpu and Vpu2-6 were normalized with respect to Tubulin concentrations (Vpu/Tubulin) to allow the comparison of Vpu and Vpu2-6 sample extracts (Vpu2-6/Vpu). (L–Q) X-Gal staining of *dpp-lacZ*/+ (L), *MS1096-Gal4/+; dpp-lacZ/+; UAS-Vpu/+* (M), *MS1096-Gal4/UAS-Vpu2-6; dpp-lacZ/+* (N), *wg-lacZ/+* (O), *MS1096-Gal4/+; wg-lacZ/+; UAS-Vpu/+* (P) and *MS1096-Gal4/UAS-Vpu2-6; wg-lacZ/+* (Q) wing imaginal discs.

The above results suggested that Vpu interacts with one or more *Drosophila* proteins thereby interfering with their normal function. Since many known roles of Vpu are due to its interaction with the human β-TrCP, we tested whether Vpu interacts with the fly β-TrCP homolog, SLIMB [33]–[35]. In human cells, the Vpu/β-TrCP interaction requires the first WD40 repeat of β-TrCP and phosphorylation of Vpu Ser52 and Ser56 [10]. Using both a yeast two-hybrid and a co-immunoprecipitation assay, we showed that Vpu interacts with the first WD domain of SLIMB, and that this interaction is abolished when using a non-phosphorylatable mutant form of Vpu, Vpu2-6, which is incapable of binding β-TrCP (Figure 2A–B).

The physical interaction between Vpu and SLIMB in *Drosophila* could explain the effects of Vpu expression through titration of endogenous SLIMB. We therefore tested the effect of expression of the Vpu2-6 mutant protein, in developing *Drosophila* wings. Surprisingly, Vpu2-6 expression led to similar adult wing defects than wild-type Vpu between veins L2 and L3, however with significantly weaker expressivity: at 24.5°C, wings of Vpu2-6-expressing flies were wild type (Figure 2C,H), while expression of Vpu induced tissue reduction between veins L2–L3 and L3–L4, proximal cross vein loss and interruption of the L3 vein (Figure 2G); at 29°C, Vpu2-6 induced loss of the proximal cross vein and strong tissue reduction between veins L2–L3 (Figure 2E), while Vpu additionally induced complete fusion of veins L2 and L3 and tissue reduction between veins L3–L4 (Figure 2D). These differences were observed for several independent transgenic lines expressing Vpu or Vpu2-6, and levels of the two proteins were shown to be equivalent in these lines (Figure 2K). Consistent with Vpu effects in the L3–L4 region, Vpu expression and *slimb* loss of function led to partially overlapping phenotypes. Indeed, Vpu expression phenocopied some previously reported effects of SLIMB depletion: ectopic expression of *dpp* (Figure 2L,M) and *wingless* (*wg*) reporter constructs (Figure 2O,P) [33], while Vpu2-6 did not (Figure 2N, Q). In addition, when *slimb* expression was reduced by RNA interference in the *dpp-Gal4* expression domain, tissue loss between veins L3 and L4, including the proximal cross vein, was observed (Figure 2F), as for Vpu expression, but the latter additionally affected the L3 and the region between veins L2 and L3 (Figure 2G). Moreover, reduction of *slimb* in the *dpp* domain did not enhance the effects Vpu expression, the resulting phenotype mainly corresponding to the addition of the two individual phenotypes (Figure 2I). Finally, *slimb* overexpression did not suppress the effects of Vpu, instead they were enhanced (Figure 2J), while overexpression of *slimb* alone in the same domain had no effect (data not shown).

Thus, despite the conservation of the Vpu/SLIMB physical interaction, our results suggest that Vpu exerts SLIMB-dependent effects between veins L3 and L4 and SLIMB-independent effects anteriorly between veins L2 and L3 in the fly wing, implicating the presence of additional Vpu partners.

### III - The Vpu-induced wing phenotype is suppressed by *thread*/DIAP1 overexpression

**Figure 3:**
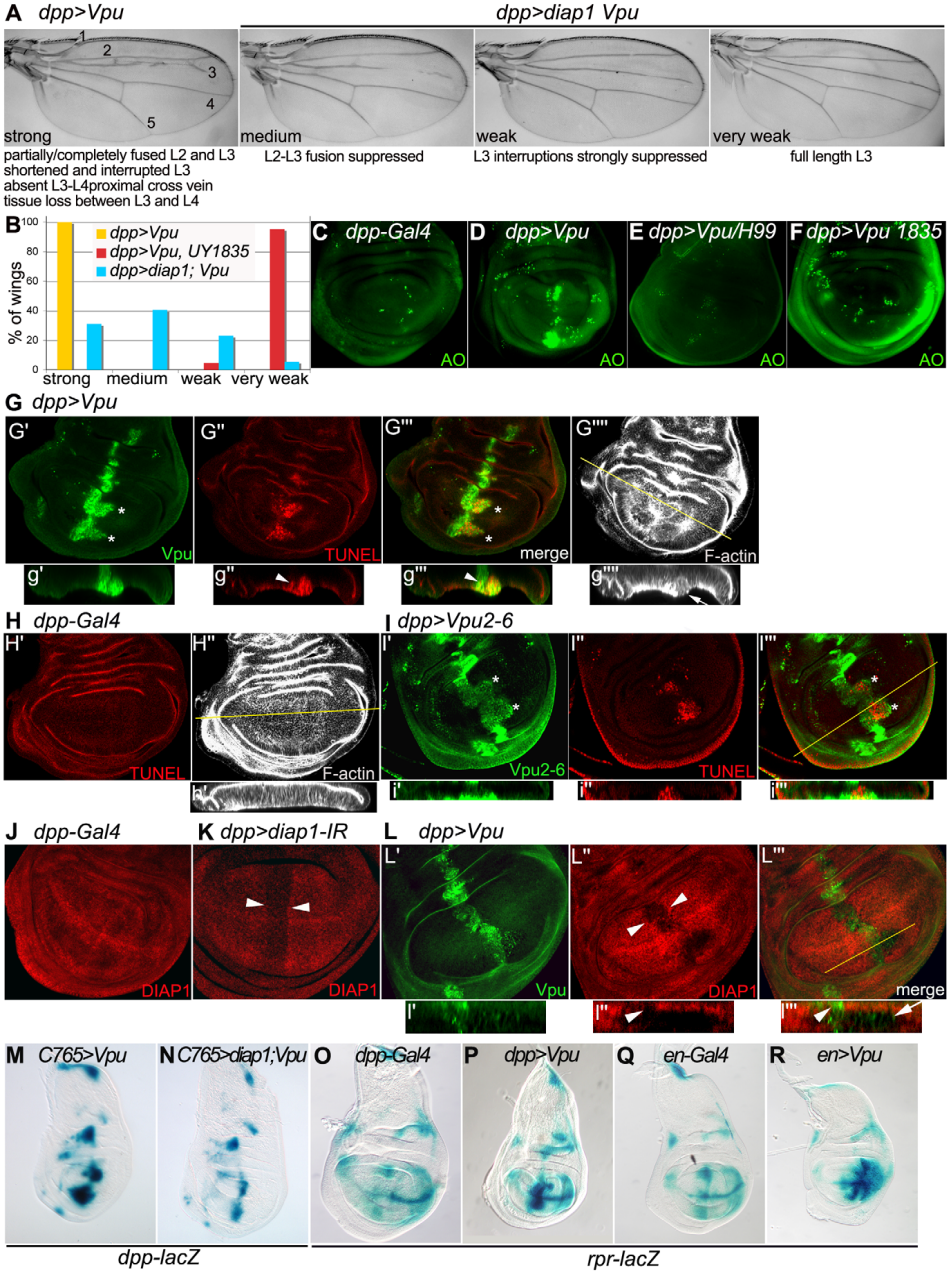
Vpu expression induces apoptosis correlated with basal extrusion of apoptotic cells in the wing disc. (A) *dpp>Vpu* wing phenotypes (26°C) suppressed to varying degrees (medium to very weak) by *diap1* overexpression (*dpp>diap1, Vpu*). (B) Distribution (% of wings) between the different phenotypic classes at 26°C. The distribution when *diap1* is over-expressed (blue and red) is statistically different in a Chi^2^ test from the reference distribution (yellow, n = 282): p = 1.4 10^−86^ with *UY1835* (n = 107) and p = 2.9 10^−51^ with *UAS-diap1* (n = 74). (C–F) Acridine Orange (AO) staining of *dpp-Gal4/+* (C), *dpp-Gal4 UAS-Vpu/+* (D), *dpp-Gal4 UAS-Vpu/Def(H99)* (E) and *dpp-Gal4 UAS-Vpu/UY1835* (F) wing discs. (G–L) XY confocal sections of wing discs. (G) *dpp-Gal4 UAS-Vpu*/+ showing Vpu immunostaining (G′; green), TUNEL staining (G″; red), merge (G′″) and F-actin network (G″″; phalloidin labeling, gray). (H) *dpp-Gal4/+* showing TUNEL staining (H′; red) and F-actin (H″; gray). (I) *Vpu2-6/+; dpp-Gal4/+* showing Vpu2-6 immunostaining (I′; green), TUNEL staining (I″; red) and merge (I′″) at 29°C. Asterisks in G′,G′″, I′,I′″: patches of Vpu-expressing cells displaced posteriorly. Below each image in G–I and L: transverse XZ section (g′-g″″, h′, i′-i″′ and I′-I′″) through the plane indicated by the line in G″″, H″ I′″, and L′″, respectively. Epithelial cells are oriented with basal towards the bottom. (g″″, h′): F-actin. Arrowhead in g″-g′″: TUNEL staining in Vpu-expressing cells not extruded from the epithelial sheet. Arrow in g″″: cells extruding from the epithelium. (J–L) Immunodetection of DIAP1 (red) and Vpu (green) in *dpp-Gal4/+* (J), UAS *diap1-IR/+; dpp-Gal4/+* (K) and *dpp-Gal4 UAS-Vpu*/+ (L) discs. Arrowheads in K and L″: DIAP1 reduction. Arrowheads and arrow in l″-l′″: DIAP1-downregulation in Vpu-expressing cells located in the *dpp* stripe and in those extruded from the epithelium, respectively. (M–N) *dpp-lacZ* expression (X-Gal staining) in *C765-Gal4/+; UAS-Vpu/+* (M) and *C765-Gal4/UAS-diap1; UAS-Vpu/+* (N). (O–R) *rpr-lacZ* expression in *dpp-Gal4/+* (O), *dpp-Gal4 UAS-Vpu/+* (P), *en-Gal4/+* (Q), *en-Gal4/+; UAS-Vpu/+* (R).

To identify novel Vpu partners, we performed a gain-of-function (GOF) genetic screen in *Drosophila*. The GOF strategy relied on a *P[yellow^+^-UAS]* transposon, whose insertion often results in Gal4-dependent upregulation of neighboring genes [36]. We screened for *P[yellow^+^-UAS]* insertions that modified the wing and eye phenotypes induced by Vpu when over-expressed in the *dpp*- and *GMR-Gal4* territories, respectively. Among 1200 lines tested, 3.8% and 4.1% enhanced the wing and eye phenotypes, respectively, while 7.3% and 1.2% suppressed the wing and eye phenotypes, respectively. We identified 51 of the modifiers genes (Table S1) and chose to further characterize one (called *UY1835*) that suppressed the effects of Vpu specifically in the wing but not in the eye (Figure 3A,B). This line corresponded to the integration of *P[yellow^+^-UAS]* in the 5′UTR of the *thread* gene. We verified that the encoded DIAP1 protein was overexpressed and fully functional (see Materials and Methods). In addition, a *UAS-diap1* construct also suppressed the effects of Vpu on the adult wing (Figure 3B). Moreover, the overexpression of *diap1* suppressed *dpp-lacZ* ectopic upregulation due to Vpu expression (Figure 3M,N). Therefore, overexpression of DIAP1 counteracts the effects of Vpu in the wing, which suggested that Vpu induces apoptosis in this tissue.

### IV - Vpu induces wing cell apoptosis associated with cell extrusion, DIAP1 downregulation and *rpr* upregulation

To test whether the loss of wing tissue induced by Vpu was due to cell death by apoptosis, we used acridine orange (AO) staining, and Terminal Transferase dUTP Nick-End Labeling (TUNEL). These two methods revealed an increase in apoptotic cell death in territories in which Vpu or Vpu2-6 were expressed (Figure 3C,D and G–I). Almost all of the TUNEL nuclear signal was located in cells with Vpu or Vpu2-6 accumulation in the cytoplasm as evidenced by co-immunostaining, suggesting that Vpu and Vpu2-6 induce cell death in a cell-autonomous manner (Figure 3G–I, g′″-i′″). Given the small size of wing disc cells, we could not address whether, as described in human cells [9], [37], Vpu localized predominantly to the perinuclear region of the cell, which includes ER, Golgi membranes and the nuclear envelope.

To confirm the pro-apoptotic effect of Vpu in *Drosophila*, we tested whether the effects of Vpu could be suppressed by downregulation of the pro-apoptotic genes *reaper (rpr)*, *grim* and *head involution defective* (*hid*) (RHG family). These genes are thought to induce apoptosis by stimulating DIAP1 auto-ubiquitylation and degradation and by repressing *diap1* mRNA translation, thereby alleviating DIAP1-dependent inhibition of downstream caspases [38]–[41]. The loss of one copy of all of these genes (due to a large deficiency *Df(3L)H99* that uncovers the three genes) was sufficient to strongly suppress the effects of Vpu expression (driven by *dpp-Gal4*) on the adult wing (leading to wild type or “very weak phenotype”, Figure 3A), as well as on cell death in the wing imaginal disc (Figure 3E). The overexpression of DIAP1 (*UY1835*) also suppressed the pro-apoptotic effect of Vpu in the wing imaginal disc (Figure 3F), which is consistent with the suppression of the adult wing phenotype (Figure 3A,B).

The *Drosophila* wing imaginal disc is a columnar pseudo-stratified monolayered epithelium (Figure 3H″, h′). Close examination of the Vpu- and Vpu2-6-expressing cells at the A/P compartment boundary within the wing pouch showed that some of them gathered into two patches positioned posterior to this boundary (Figure 3G′,G′″ and I′,I′″, asterisks) that likely correspond to the enlarged areas of the *dpp-lacZ* stripe in Figure 1G (asterisks). The cells within these patches expressed Vpu or Vpu2-6 and underwent apoptosis. Virtual sections along the apico-basal axis (X-Z focal planes) revealed that Vpu- and Vpu2-6-expressing apoptotic cells were misplaced posteriorly to the *dpp*-expression domain and were extruded basally from the wing disc epithelium (Figure 3g′-g′″ and i′-i′″), which was altered with respect to F-actin organization and exhibited multilayering of cells (arrow in Figure 3g″″). TUNEL staining was also detected in some Vpu-expressing cells that were present within the *dpp* expression stripe and properly positioned within the epithelium (Figure 3g″ and g′″, arrowheads). Altogether, these results demonstrated that in *Drosophila*, as in human cells, Vpu expression induces apoptotic cell death, thereby providing us with a model system for identifying cellular partners and signaling pathways recruited by Vpu in this process *in vivo*.

Given that the pro-apoptotic effects of Vpu were suppressed by overexpression of DIAP1, an attractive hypothesis was that Vpu pro-apoptotic effects might be due to downregulation of the DIAP1 protein. We therefore monitored the levels of DIAP1 in the wing imaginal disc: Vpu expression at the A/P compartment boundary led to a decrease in DIAP1 accumulation in the same region (Figure 3J,L arrowheads; similar to that induced by downregulation of DIAP1 by RNA interference Figure 3K, arrowheads), that is even more pronounced in Vpu-expressing cells posteriorly positioned and extruding. This result reinforces the hypothesis that cell extrusion is a consequence of apoptosis.

The pro-apoptotic proteins RPR, HID, and GRIM induce apoptosis by antagonizing DIAP1 function [42]. We therefore monitored the effect of Vpu on *rpr* and *hid* expression levels using *lacZ* reporters. Robust upregulation of *rpr-lacZ* (but not *hid-lacZ*) expression was found in the Vpu expression domain (Figure 3O–R and data not shown), indicating that Vpu promoted *rpr* transcription. Taken together, our results strongly suggest that Vpu induces apoptosis via *rpr* upregulation and DIAP1 downregulation.

### V - Caspase activity is necessary for Vpu-induced cell death in the *Drosophila* wing

**Figure 4:**
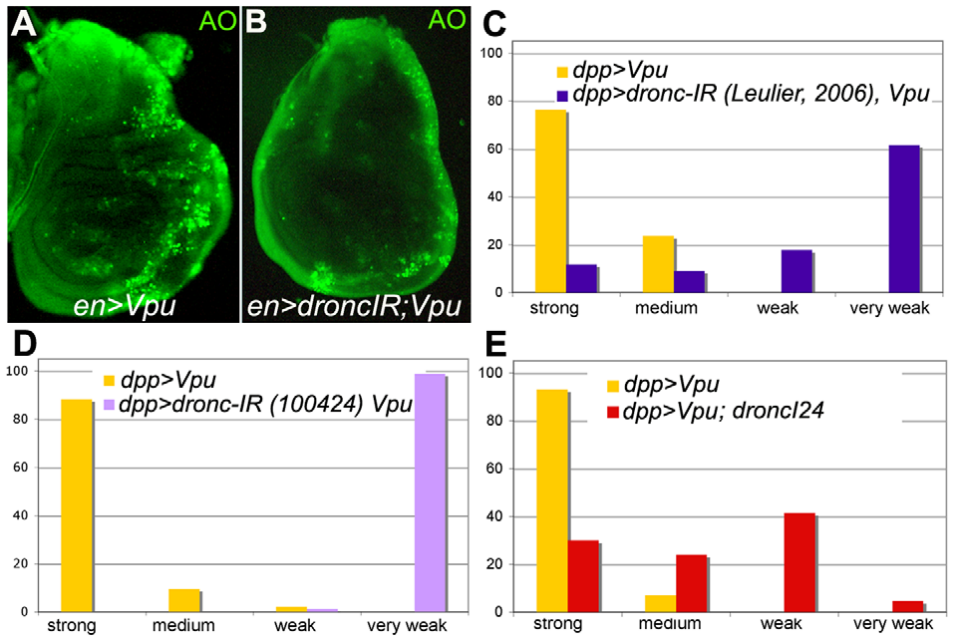
Vpu-induced apoptosis requires DRONC caspase activity. (A–B) Acridine Orange (AO) staining of *en-Gal4/+; UAS-Vpu/+* (A) and *en-Gal4/UAS-dronc-IR; UAS-Vpu/+* (B) wing imaginal discs. (C–E) The distribution of wing phenotypes induced by *dpp-Gal4*-driven expression of Vpu when *dronc* is down-regulated is statistically different (Chi^2^ test) from the reference distribution (C–E, yellow; n = 55, n = 137, n = 230, respectively): p = 1.7 10^−30^ with *UAS-dronc-IR* [83] (C; n = 299), p = 1.6 10^−42^ with *UAS-dronc-IR* (VDRC line 100424) (D; n = 87) and p = 8.2 10^−44^ with *dronc^I24^*/+ (E; n = 217).

To determine whether Vpu-induced cell death was dependent on caspase activity, we tested the effect of reducing the levels of the initiator caspase Dronc. We found that Vpu-induced cell death was partially suppressed as evidenced by AO staining (Figure 4A,B) and by the adult wing phenotype (Figure 4C–E). Vpu-induced cell death thus depends on *dronc* function.

**Figure 5:**
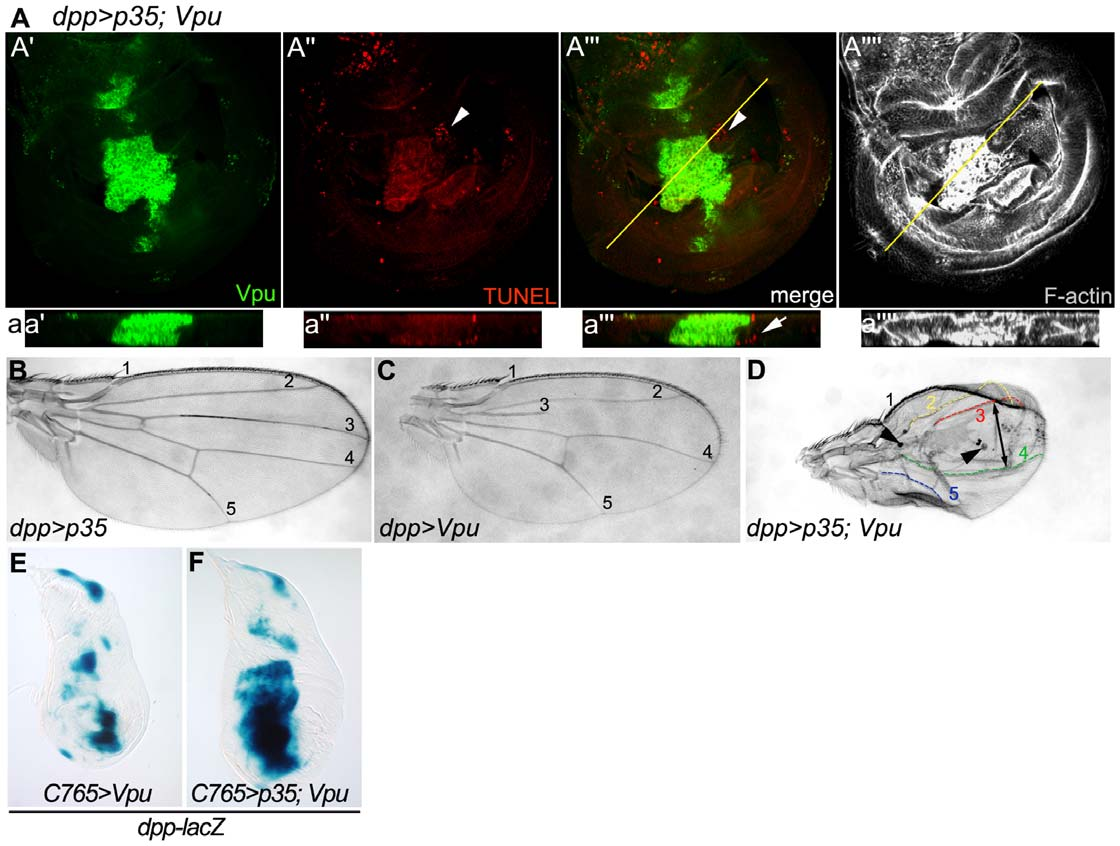
Inhibitor of caspase activity P35 suppresses Vpu-induced apoptosis in the developing wing. (A) Confocal XY section showing Vpu immunostaining (A′; green), TUNEL staining (A″; red), Vpu and TUNEL merge (A′″) and F-actin labeling (A″″; gray) in a *UAS-p35/+; dpp-Gal4 UAS-Vpu/+* wing disc. (a) Transverse XZ sections through the planes indicated by the line in A′″ and A″″. Arrowhead in A″, A′″ and arrow in a′″: apoptosis outside of the Vpu-expression domain. (B–D) Adult wings from *UAS-p35/+; dpp-Gal4/+* (B), *dpp-Gal4 UAS-Vpu/+* (C) and *UAS-p35/+; dpp-Gal4 UAS-Vpu/+* (D) flies. In D, longitudinal veins are outlined with a yellow (L2), red (L3) green (L4) and blue (L5) dotted line, respectively. Note the restoration of the L3 vein, the absence of fusion between L2 and L3, the expansion of the space between L3 and L4 (arrow) and the exclusion of patches of cells in the wing blade (arrowheads). (E–F) *dpp-lacZ* expression (X-Gal staining) in *C765-Gal4/+; UAS-Vpu/+* (E) and *C765-Gal4/UAS-p35; UAS-Vpu/+* (F).

To further investigate the requirement of caspases for Vpu-induced cell death, we tested the effect of P35, a baculovirus protein known to block effector caspase activity (reviewed in [43], [44]). Although the adult wing appears broadly disorganized (see Figure 5D), co-expression of P35 and Vpu at the A/P boundary completely suppressed apoptosis in Vpu-expressing cells as determined by reduced TUNEL staining (Figure 5A″ compare to Figure 3G″), which is correlated with the recovery of a full length L3 vein and the partial restoration of tissue between veins L2 and L3 in the adult wing (Figure 5B–D). Therefore, Vpu-induced phenotypes are caspase-dependent.

However, co-expression of P35 and Vpu resulted in additional phenotypes compared to the expression of Vpu alone. An expansion of the space between veins L3 and L4 was observed (Figure 5D, arrow), which is in accordance with the widening of the Vpu-expression domain in the wing disc (Figure 5A′, compare to Figure 3G′,L′). In the same region, the epithelial sheet was very disorganized, displaying several folds (Figure 5A″″, a″″). Vpu-expressing cells might thus be kept alive by concomitant expression of P35, leading to an increased accumulation of these cells at the A/P boundary. Surprisingly, the overall size of the wing was reduced (Figure 5D) which perhaps can be attributed to the apoptosis detected outside of the Vpu-P35-expression domain in the wing disc (Figure 5A″,A′″, arrowhead and 5a, arrow). Finally, in the adult wing, patches of cells seem to be excluded from the wing epithelium (Figure 5D, arrowheads), possibly as a consequence of over-proliferation of cells of the wing disc epithelium. In fact, previous characterization of cells targeted to death in which P35 expression blocks cell death (termed “undead cells”) has shown that these cells induce the hyper-proliferation of neighboring cells [45]–[47] *via* secretion of DPP and WG [48]. We tested whether the co-expression of P35 with Vpu led to the accumulation of cells expressing *dpp*. We found that the ectopic expression of *dpp* resulting from Vpu expression is dramatically increased when P35 is co-expressed (Figure 5E–F), suggesting that “undead” cells expressing Vpu might induce over-proliferation of neighboring cells through the prolonged secretion of the *dpp* growth factor.

### VI - The JNK pathway is activated in Vpu-expressing cells undergoing apoptosis

**Figure 6:**
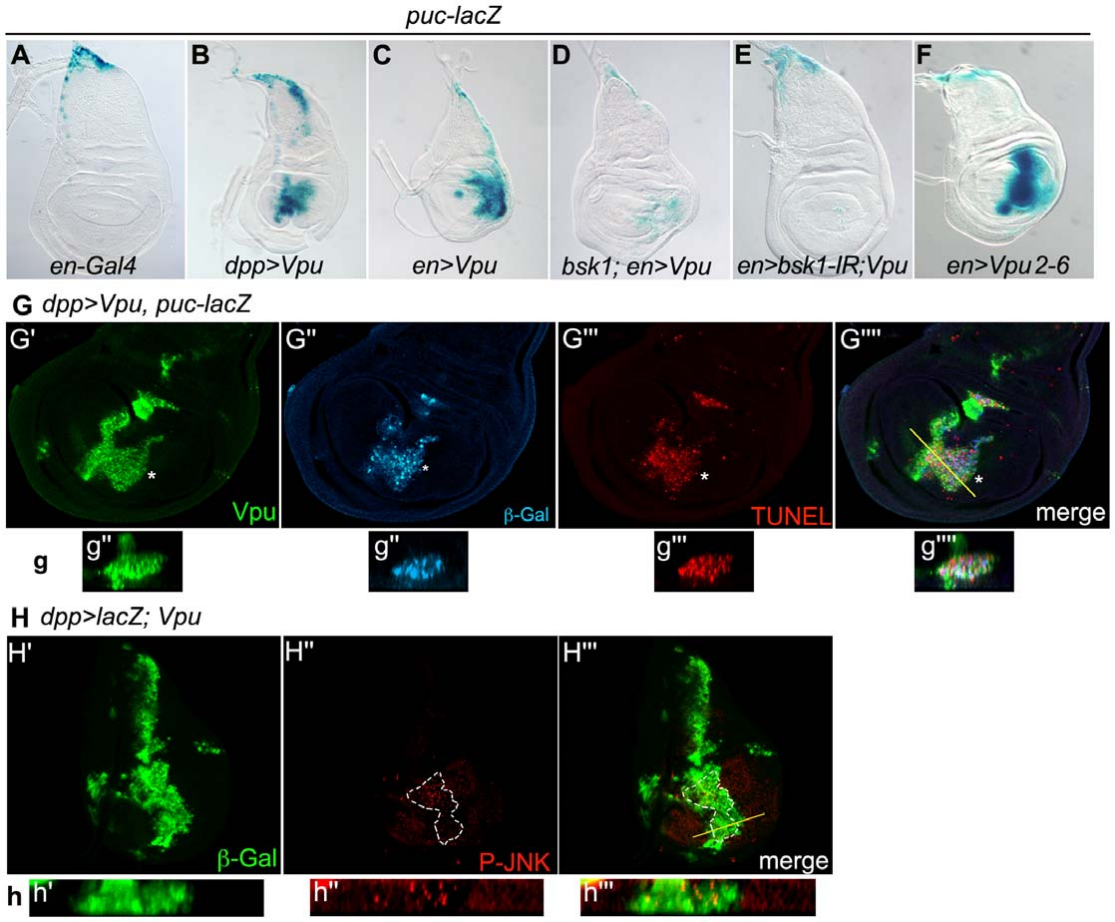
The JNK pathway is activated in apoptotic cells expressing Vpu. (A–F) *puc-lacZ* expression (X-gal staining) in *en-Gal4/+* (A) *dpp-Gal4 UAS-Vpu/+* (B), *en-Gal4/+; UAS-Vpu/+* (C), *en-Gal4/bsk^1^; UAS-Vpu/+* (D), *en-Gal4/+; UAS-bsk-IR UAS-Vpu/+* (E) and *Vpu2-6/+; en-Gal4/+* (F) wing imaginal discs. In control discs (A), as in wild type wing discs, *puc-lacZ* expression was restricted to the tip of the notum. (G) *dpp-Gal4 UAS-Vpu/puc-lacZ* wing disc XY confocal section showing Vpu (G′; green) and β-galactosidase (G″; blue) immunostaining, TUNEL labeling (G″′; red) and the merge (G″″). The induction of *puc-lacZ* expression was restricted to the wing pouch (G″). Asterisks in G′-G″″: patch of dying cells expressing both Vpu and *puc-lacZ*, located posteriorly from the *dpp* expression stripe. (H) Immunodetection of β-galactosidase reporting on the Vpu expression domain (H′; green), phosphorylated Basket (P-JNK) (H″; red) and merge (H′″) in a *UAS-lacZ/+; dpp-Gal4 UAS-Vpu/+* wing imaginal disc. The territory of P-JNK staining is delimited with a dotted line (H″, H″′). Below each image in G,H: transverse XZ section through the plane indicated by the line in G″″ and H′″, respectively. All the extruding cells and some of the cells in the *dpp* stripe co-express Vpu and *puc-lacZ* (g). The wing imaginal disc shown in H is from a young third instar larva in contrast to most of the other wing discs presented which are from wandering third instar larvae.

Our results indicate that Vpu-induced apoptosis in the wing is correlated with both *rpr* induction and DIAP1 downregulation. Several reports have established a connection between DIAP1, RPR and the JNK pathway and suggest that these proteins may be part of a regulatory loop (see discussion). Ectopic activation of the JNK pathway is known to have a pro-apoptotic effect in the *Drosophila* wing disc [49]–[52]. In addition, in this same tissue, *rpr* is a transcriptional target of the JNK pathway in response to stress conditions [53] and ectopic expression of *rpr* can promote DIAP1 degradation, which in turn activates the JNK pathway [54]. We thus decided to test whether Vpu expression has an effect on JNK pathway activation in the wing imaginal disc. *puckered* (*puc*), encoding a Jun kinase phosphatase, is a transcriptional target of the JNK signaling pathway and acts in a negative feedback loop to dampen JNK signaling [55]. To analyze *puc* expression, we used the *puc-lacZ* transcriptional reporter (*puc^E69^* allele) known to be a consistent read-out of JNK activation and to result in modest upregulation of JNK signaling (Figure 6). When Vpu was expressed in the *dpp* or in the *en* expression domains, ectopic *puc-lacZ* expression was detected in the corresponding domains (Figure 6A–C and 6G′,G″). Strikingly, the activation of *puc-lacZ* was especially strong in the TUNEL-positive Vpu-expressing cells displaying posterior displacement with respect to the *dpp* domain and basal extrusion (Figure 6G,g). In this *puc-lacZ/+* heterozygous background, the effects of Vpu in the wing were enhanced: induction of apoptosis (Figure 6G′″, compare to 3G″), deformation of the wing discs (Figure 6B,C compare to 1G,E), fusion of wing veins L2 and L3 and reduction of the wing blade (data not shown). Vpu2-6 also activated the JNK pathway (Figure 6F).

The activation of the JNK pathway by Vpu was further analyzed by assaying the phosphorylation state of the *Drosophila* JNK, Basket (BSK/DJNK, [56]) in wing imaginal discs using an anti-phospho-JNK (JNK-P) antibody. In cells of the wing pouch expressing Vpu, phosphorylated JNK was observed (Figure 6H,h). Taken together, these results indicate a correlation between Vpu-induced cell death and activation of the JNK pathway.

### VII - Vpu-induced apoptosis in the wing disc requires BSK/DJNK function

**Figure 7:**
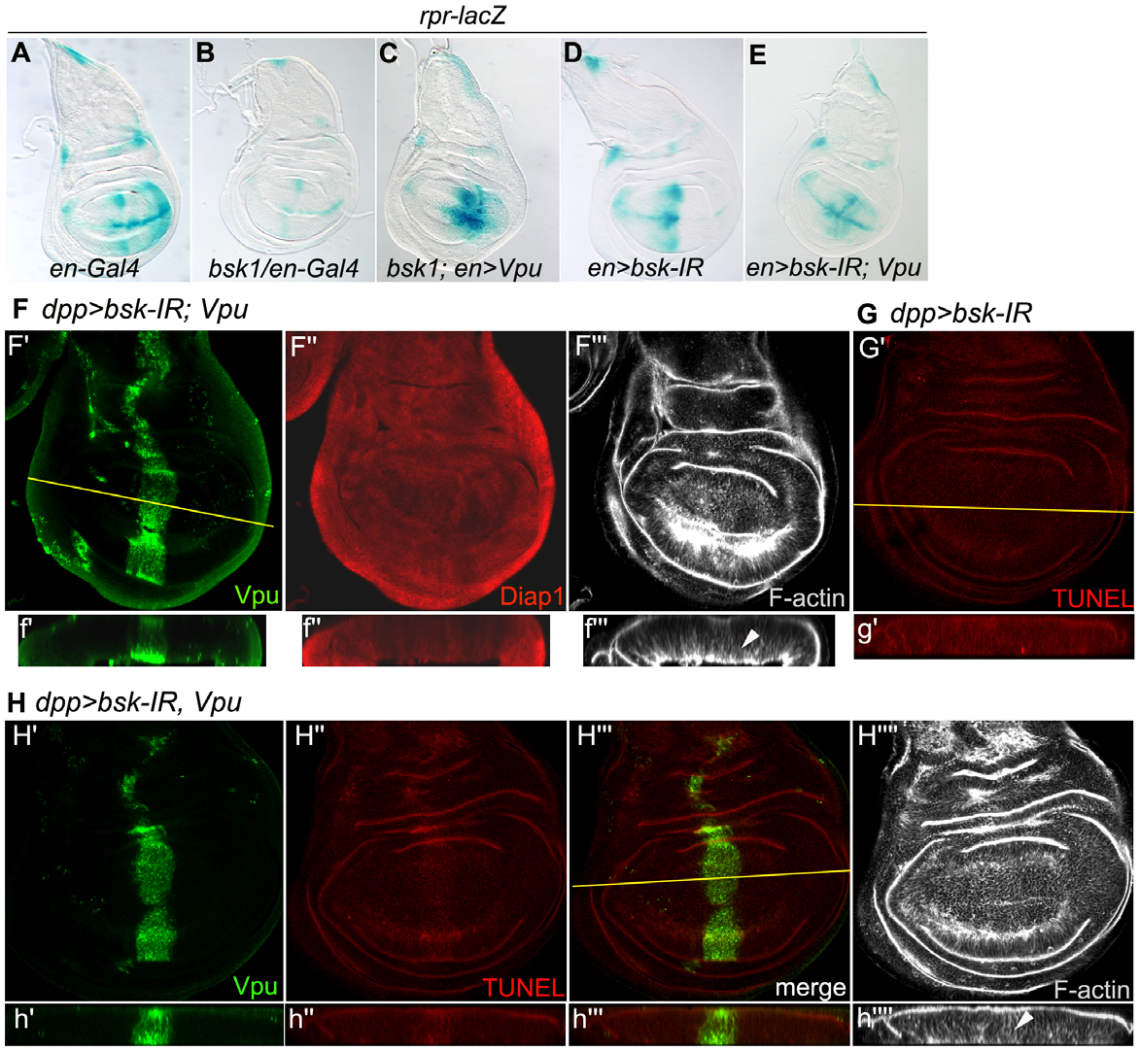
Vpu-induced *rpr* upregulation, DIAP1 downregulation and apoptosis in the wing disc depend on JNK pathway activity. (A–E) *rpr-lacZ* expression (X-gal staining) in *en-Gal4/+* (A) *bsk^1^/en-Gal4* (B), *bsk^1^/en-Gal4; UAS-Vpu/+* (C), *en-Gal4/+; UAS-bsk-IR/+* (D) and *en-Gal4/+; UAS-Vpu UAS-bsk-IR*/+ (E). (F) XY confocal section of Vpu (green), DIAP1 (red) immunostaining and F-actin network (gray) of a *dpp-Gal4/UAS-bsk IR UAS Vpu* wing imaginal disc. (G–H) XY confocal section of Vpu immunodetection (H′,H; green), TUNEL labeling (G′,H″,H′″; red) and F-actin network (H″″; gray) of *dpp-Gal4/UAS-bsk-IR* (G) and *dpp-Gal4/UAS-Vpu UAS-bsk-IR* (H) wing imaginal discs. Below each image in F–H: transverse XZ section (f′-f′″, g′ and h′-h″″) through the plane indicated by the line in F′, G′ and H′″, respectively. The epithelial sheet is not disorganized (arrowheads in f′″ and h″″) by Vpu-expression when *bsk* is concomitantly knocked-down by *RNAi*.

**Figure 8:**
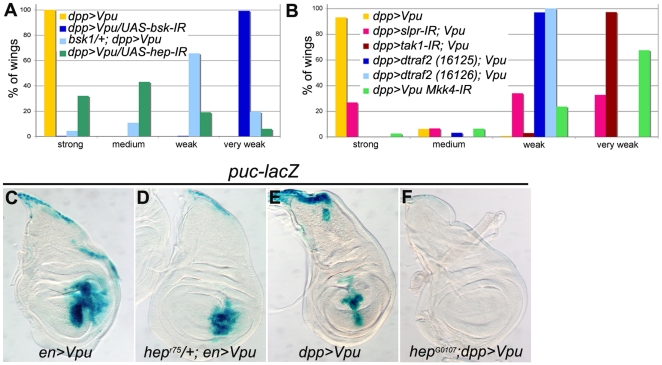
Vpu activates the JNK pathway in wing cells upstream of *hep*. (A) Phenotypic distributions (as %) of wings of the indicated genotypes. The distributions observed when *bsk* (dark and light blue) or *hep* (green) are down-regulated are statistically different (Chi^2^ test) from the reference distribution (yellow, n = 282): p = 7.8 10^−112^ with *UAS-bsk-IR* (n = 223) and p = 1.2 10^−56^ with *UAS-hep-IR* (n = 187), and (yellow n = 403): p = 1.5 10^−90^ with *bsk^1^*/+ (n = 93), respectively. Flies were raised at 26°C. (B) Phenotypic distribution (as %) of wings of the indicated genotypes. Three independent experiments are shown but, in each case, the phenotypic distribution of wings in the control (*, **, *** see below) is identical for each set of experiments. The distributions observed with (1) RNAi-mediated knockdown of *slp* and *tak1* (pink and brown, respectively) are each statistically different (Chi^2^ test) from the reference distribution (yellow*; n = 222 and n = 220, respectively): p = 8.2 10^−43^ with *UAS-slpr-IR* (n = 168) and p = 4.3 10^−82^ with *UAS-tak1-IR* (n = 169), (2) two *UAS-traf2-IR* insertions are statistically different from the reference distribution (yellow**; n = 258): p = 1,9 10^−87^ with *UAS-traf2-IR* (light blue VDRC16125; n = 159) and p = 5,3 10^−72^ with *UAS-traf2-IR* (dark blue VDRC16126; n = 64), (3) RNAi-mediated knockdown of *Mkk4* (green, n = 267) is statistically different from the reference distribution (yellow***; n = 251): p = 1,4 10^−96^. (C–F) *puc-lacZ* expression (X-gal staining) in wing imaginal discs of *en-Gal4/+*; *UAS-Vpu/+* (C) and *hep^r75^/+; en-Gal4/+; UAS-Vpu/+* (D) females and of *dpp-Gal4 UAS-Vpu/+* (E) and *hep^G0107^/Y; dpp-Gal4 UAS-Vpu/+* (F) males. Larvae were raised at 22°C since the *hep^G0107^/Y* mutant is lethal before the third larval instar at a higher temperature.

To address whether Vpu-induced cell death depends on the JNK pathway, we tested whether BSK/DJNK, which plays a central role in the activation of the JNK pathway, was required for the different effects of Vpu that we observed in the wing. In wing discs expressing Vpu in the *en* domain, we reduced the dose of *bsk* by using either a heterozygous context for a null mutant allele, or a *UAS-bsk-IR* construct. We found that both *bsk* mutant contexts were associated with a decrease in *rpr-lacZ* basal expression in the wing disc (Figure 7B,D, compare to 7A), consistent with results from a previous report [53]. Strikingly, Vpu-induced *rpr-lacZ* expression was strongly reduced in the context of diminished *bsk* activity (Figure 7C,E compare to 3R), and that of *puc-lacZ* almost completely abolished in this same context (Figure 6C–E). These results show that Vpu activates expression of both the *rpr* and *puc* promoters *via* the JNK pathway and not by direct transcriptional regulation. Reduction of *bsk* activity also completely suppressed Vpu-induced downregulation of DIAP1 (Figure 7F″, compare to 3L″) and almost completely suppressed apoptosis (Figure 7H compare to 7G and 3G). It is noteworthy that when Vpu was co-expressed with *bsk-IR* under the control of *dpp-Gal4*, the Vpu expression domain became enlarged when compared to control discs expressing Vpu alone (compare Figure 7F′ and H′ to Figure 3G′). This result may be explained by the concomitant suppression of the posterior displacement (Figure 7F′ and H′ compare to Figure 3G′″, asterisks), basal extrusion (Figure 7f′-f′″,h′-h″″ compare to Figure 3g″″) and apoptosis of Vpu expressing cells observed when *bsk* was downregulated. Finally, *bsk* downregulation strongly suppressed the Vpu-induced wing phenotype (Figure 8A).

Altogether, these results demonstrate that all the effects induced by Vpu both in the wing disc and in the adult wing require the activity of *bsk* and therefore depend on the activity of JNK pathway. Importantly, the activation of *rpr*- and *puc-lacZ* resulting from Vpu expression was not suppressed when P35 was co-expressed with Vpu (Figure S2). Thus, neither Vpu-mediated activation of the JNK pathway, nor that of *rpr* expression, is dependent on caspase activity. This reinforces the above conclusion that Vpu-induced apoptosis is mediated by the activation of the JNK pathway.

### VIII- Other JNK pathway components are also involved in Vpu-induced wing phenotypes

Our results showed that Vpu activates the JNK pathway upstream of, or through, *bsk*, which, in turn, induces the apoptosis cascade. To characterize more precisely the target(s) through which Vpu activates the JNK pathway, we tested the effect of the loss of function of several regulators of the JNK pathway on the Vpu-induced wing phenotypes. We first tested *hemipterous* (*hep*) which encodes a JNK kinase (JNKK) acting upstream of DJNK/BSK. Downregulation of *hep* (*UAS*-*hep-IR*) suppressed the effects of Vpu on the adult wing (Figure 8A). Accordingly, Vpu-induced *puc-lacZ* expression was reduced in a *hep* heterozygous mutant background (Figure 8C,D) while it was totally abolished in a *hep* hemizygous mutant background (Figure 8E,F). Suppression of the wing phenotype induced by Vpu was also obtained when two of the JNKKKs known to activate the Hep-Bsk cascade were downregulated: *dTAK1* (the TGF-beta-activated kinase 1/MAPK3; [57]) and the *MLK/Slipper* (*Slpr*; [58], [59]) using *UAS*-*dTak1-IR* or *UAS*-*slpr-IR* constructs, respectively (Figure 8B). We also tested intracellular proteins known to activate JNKKKs in response to various stimuli (reviewed in [60]) such as the Tumor-Necrosis Factor Receptor-associated factor-1 (DTRAF1, the TRAF2/6 ortholog; [54]), the Ste-20 related kinase Misshapen (MSN, related to GCK/PAK; [61]), DTRAF2 (the TRAF6 ortholog [62]), DRac1 (a small GTPase of the Rho family; [63]) and the only two known *Drosophila* homologues of the TNF/TNFR family members, Eiger (EGR) and Wengen (WGN), respectively; [64]–[67]. We tested these candidates by down-regulating their expression either by RNA interference (*UAS-dtraf1-IR*, *UAS-dtraf2-IR*, *UAS-msn-IR*, *UAS*-*eiger-IR* and *UAS*-*wengen-IR*) or in heterozygous mutant contexts (*msn^102^*, *msn^6146^*, *Rac1^j11^* and *Rac1^j11^ Rac2^Δ^*). Among these, only the RNAi construct targeting the adaptor protein DTRAF2 suppressed the Vpu-induced wing phenotypes (Figure 8B). Taken together, our results clearly show that Vpu-induced apoptosis is mediated by the activation of the JNK pathway involving the Hep/JNKK-Bsk cascade. In addition, they suggest that Vpu-activation of this cascade occurs upstream of or through dTAK1 and Slipper, and possibly upstream of or through DTRAF2.

## Discussion

While most of the data concerning Vpu and its cellular partners come from cellular and biochemical assays, the present work validates the use of *Drosophila* to study the effects of Vpu at the level of a whole organ and to identify functional partners of Vpu *in vivo*. It sheds new light on the relationship between Vpu and apoptosis and leads to the identification of a first functional link between Vpu and JNK pathway activity, elucidating a novel way by which Vpu disturbs a host cell leading to its death.

### I – Vpu induces cell death in the developing wing

Our data show that Vpu expression in the developing fly wing disturbs its development at least in part by promoting cell-autonomous caspase-dependent apoptotic cell death. In cultured HIV-1-infected T cells and in Vpu-expressing Hela cells, Vpu was previously shown to contribute significantly to caspase-dependent apoptosis through its inhibition of I-κB degradation [20], [21]. This pro-apoptotic effect of Vpu was shown to involve its interaction with β-TrCP. Likewise, in human HIV-1 infected T cells and in immortalized cell lines transfected with Vpu-expressing constructs, Vpu promotes p53-mediated apoptosis in a β-TrCP-dependent manner [23]. Our results demonstrate that Vpu also interacts physically with fly SLIMB/β-TrCP. However, several lines of evidence indicate that the pro-apoptotic effects of Vpu in the fly wing are at least partly independent of the interaction of Vpu with SLIMB/β-TrCP. In fact, 1) expression of Vpu2-6 induces a phenotype only detectable between veins L2 and L3 of the wing (Figure 2E compare to 2C, purple arrow), qualitatively similar to that resulting from Vpu expression, but significantly weaker, 2) expression of Vpu2-6 also induces apoptosis and activates the expression of *puc-lacZ* in the wing imaginal disc, showing that the inability of Vpu2-6 to interact with SLIMB does not abolish its apoptogenic properties, and 3) downregulation of *slimb* in the *dpp* domain of the wing mimics the effects of Vpu expression between L3 and L4 veins but not between L2 and L3. Taken together, our data suggest that Vpu induces apoptosis in *Drosophila* wing cells *via* at least two mechanisms: 1) a SLIMB/β-TrCP-independent mechanism and 2) a SLIMB/β-TrCP -dependent mechanism which could explain the much stronger effects always obtained with Vpu compared to those with Vpu2-6. In both cases, Vpu-induced apoptosis is strictly dependent on JNK pathway activity since it is fully abrogated in a *bsk* mutant background.

Although Vpu β-TrCP-dependent effects in human cells were previously shown to be due to titration of endogenous β-TrCP [20], [23], we found, unexpectedly, that overexpression of SLIMB in Vpu-expressing wing cells enhanced Vpu effects. This result therefore confirmed that a functional interaction between the two proteins occurs *in vivo*. Since endogenous levels of SLIMB in *Drosophila* wing imaginal disc cells are low (B. Limbourg-Bouchon, unpublished results), as is the case for β-TrCP in human cells [68], the overexpression of SLIMB along with Vpu might lead to the formation of abundant Vpu/SLIMB complexes thereby leading to titration of SCF ubiquitin ligase complex components such as SkpA [69], and giving rise to additional deleterious effects. Our results with *slimb* overexpression do not exclude that Vpu effects in *Drosophila* wing, in particular between veins L3 and L4, could depend on endogenous SLIMB titration, but the strong additional effects resulting from Vpu and SLIMB co-expression might mask putative suppressor effects of SLIMB.

If Vpu SLIMB/β-TrCP-dependent effects are due to titration of endogenous SLIMB, decreasing the level of endogenous SLIMB should enhance Vpu effects between veins L3 and L4. However, in a *slimb* mutant background (*slimb^0^*/+ giving rise to wild type wings (data not shown) or RNAi mediated knock-down of *slimb*), the wing phenotype between L3 and L4 veins, due to Vpu expression in the *dpp* domain, was not clearly different from that observed in a *slimb^+^* background. This might indicate that in a wild type background exogenous Vpu is not limiting and titrates all SLIMB. Thus a decrease of endogenous SLIMB would not enhance Vpu effects that are SLIMB/β-TrCP-dependent.

Analysis of the reduced, disorganized, rough eye phenotype induced by Vpu expression during development, suggests that Vpu exerts different effects in this organ. Indeed, Vpu effects in the eye were not suppressed either when the dosage of pro-apoptotic genes was reduced or when DIAP1 was co-expressed with Vpu, and were not associated with JNK activation nor *rpr* gene upregulation (data not shown). In addition, in the genetic screen for modifiers of the Vpu-induced wing and eye phenotypes, only 11% of the modifiers identified affected both tissues. Such differences between Vpu effects in the eye and wing may reflect the presence of distinct tissue-specific partners of Vpu or may be due to differences in the proliferative status of the cells in which Vpu is expressed, *i.e.* mitotic in the wing disc and post-mitotic in the eye disc. Nonetheless, our results indicate that, in *Drosophila*, Vpu effects appear to be at least in part independent of SLIMB/β-TrCP in both the eye and wing. In addition, Vpu activation of the Toll pathway upon fungal infection in the adult fly was shown to be dependant on the presence of the Vpu domain allowing interaction with SLIMB/β-TrCP, but independent of *slimb* function [31]. This suggested that Vpu exerts its effects on the immune response by binding to another as yet uncharacterized homolog of β-TrCP. The study of Vpu effects in several *Drosophila* organs and the identification of tissue-specific effects therefore increase the panel of potential Vpu functional partners.

### II – Vpu wing phenotypes result from cell-autonomous caspase-dependent apoptosis that triggers non-autonomous tissue loss

Our results demonstrate a direct association between Vpu-induced phenotypes and caspase activation in the wing epithelium. Although Vpu- and Vpu2-6-induced apoptosis in the wing disc was largely cell-autonomous, non cell-autonomous effects were also observed when Vpu and Vpu2-6 expression are driven with *dpp-Gal4*: reduction of the anterior compartment of the wing disc, additional tissue loss extending anteriorly beyond the *dpp* expression domain (between veins L2 and L3) and a global decrease of the wing size. These phenotypes might be due to the apoptosis-induced loss of *dpp*-expressing cells that would subsequently lead to an overall decrease in the DPP (the fly TGFβ homologue) morphogen in the wing disc. Interestingly, the downregulation of *slimb* in the same domain only led to cell-autonomous effects in the adult wing (*i. e.*, between veins L3–L4), suggesting that cell-autonomous Vpu effects are dependent of SLIMB, while non cell-autonomous effects are independent of SLIMB.

Interestingly, although suppression of Vpu-induced apoptosis is obtained either with co-expression of P35 or DIAP1, or with downregulation of *dronc*, resulting in partial restoration of L2–L3 inter-vein tissue and L3 length, only P35 co-expression induces an enlargement of the domain between L3 and L4, and overgrowths in the adult wing. This difference may be due to the fact that DIAP1 overexpression and *dronc* depletion block cell death upstream of caspase activation, while P35 blocks the function but not the activation of effector caspases and as such leads to the production of “undead cells” with persistent DPP/Wingless mitogen factor signaling, causing hyperplastic overgrowth [48]. In fact, when Vpu and P35 are co-expressed, *dpp-lacZ* is strongly upregulated, which might induce over-proliferation of neighboring cells. In contrast, DIAP1 overexpression suppresses Vpu-induced ectopic *dpp-lacZ* expression consistent with lack of accompanying overgrowth phenotypes.

In the absence of P35 expression, we also observed ectopic *wg* and *dpp* expression as a consequence of Vpu expression though at much lower levels (Figures 1G, 2M,P and 3M). This might be interpreted to be a consequence of either SLIMB depletion or Vpu-induced JNK pathway activation. In fact, in normal apoptotic cells, ectopic activation of *wg* and *dpp* signaling was shown to be a side effect of JNK pathway activation and not a consequence of apoptosis [48], [70]. However, the residual ectopic expression of *dpp-lacZ* still observed upon coexpression of Vpu and DIAP1, might reflect a titration of endogenous SLIMB by Vpu.

### III – Vpu-induced wing defects require activation of the JNK pathway, upstream of JNKKKs

**Figure 9:**
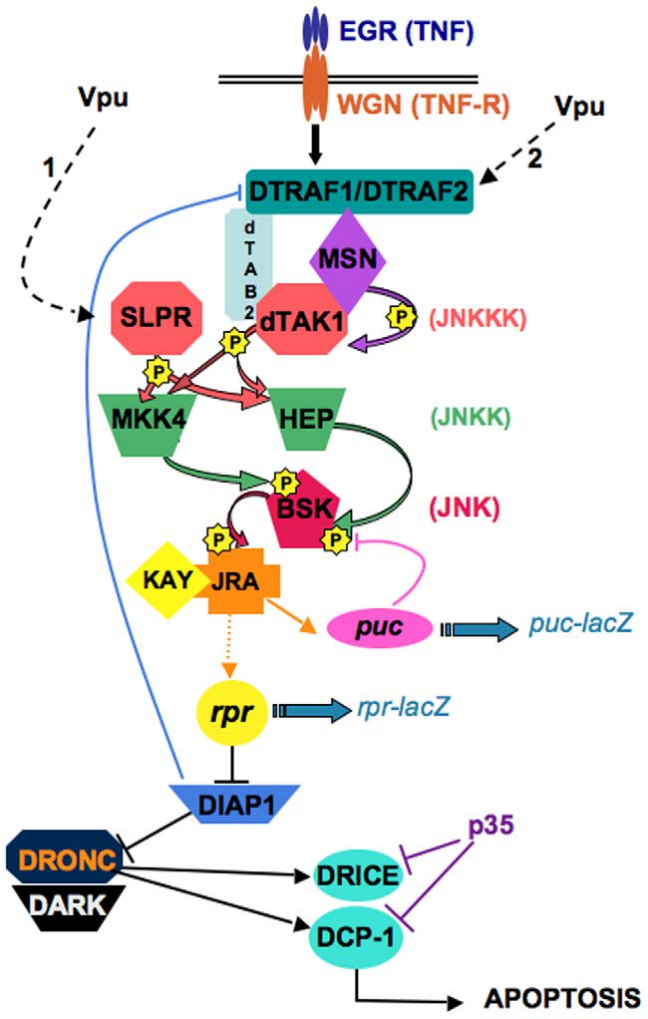
Model: Vpu activates the JNK pathway upstream of JNKKKs. The JNK pathway is a kinase cascade that, when activated in *Drosophila*, leads to phosphorylation of the transcription factors JRA (Jun-related antigen) and KAY (Kayak). *puckered* (*puc*) is a transcriptional target of the JNK pathway and acts in a negative feedback loop to dampen JNK signaling. The activation of this cascade also leads to the transcriptional activation of the pro-apoptotic gene *rpr* that in turns leads to the degradation of the anti-apoptotic factor DIAP1, which activates caspases such as DRONC/Caspase 9 and its specific co-factor DARK/Apaf-1, DRICE/Caspase 3 and Death Caspase-1 (DCP-1) resulting in the onset of apoptosis. Although *reaper* does not have a direct human homolog, mammalian Smac/DIABLO was shown to be a functional homolog of Reaper/Hid/Grim, that acts by reducing c-IAP protein levels through the ubiquitin/proteasome pathway [92]. Two JNKKs, HEP and MKK4, phorphorylate the JNK/BSK at different sites. These are phosphorylated by several *Drosophila* JNKKKs, among which SLPR and dTAK1, the latter being a target for phosphorylation by MSN. DTRAF1 and DTRAF2 are intracellular proteins known to activate JNKKKs. TAK1-associated binding protein 2 (dTAB2) is an adaptator protein linking dTRAF1 to dTAK1. The ligand-receptor interaction between EGR/WGN (TNF/TNFR) has been shown to induce JNK pathway-mediated apoptosis. Yellow stars: phosphorylation activity of the kinases. Blue, pink and violet lines: negative regulators (DIAP1 overexpression, and PUC and P35 expression negatively regulate DTRAF1, BSK and effector caspase activities, respectively). Wide blue arrows: *lacZ* reporter transgenes. The dotted arrows 1 and 2 indicate the candidate targets of Vpu suggested by this work.

Our results demonstrate that Vpu-induced wing defects depend on the function of specific components of the JNK pathway such as BSK/JNK and the HEP/JNKK. In particular, in the wing, our results suggest that Vpu acts upstream of or at the level of both JNKKKs, DTAK1 and SLPR (Figure 9, dotted arrow 1). These two gene functions are also necessary for the JNK pathway-dependent apoptosis resulting from overexpression of the Rho1 GTPase in the wing [52]. The authors also found that DTAK1 co-immunoprecipitated with SLPR and Rho1, and proposed that a large protein complex may form for activation of the JNK pathway. Our results suggest that Vpu may activate these JNKKKs *via* DTRAF2 (Figure 9, dotted arrow 2). DTRAF2 acts as an adaptor protein through which tumor suppressor dCYLD has been shown to regulate TNF-induced JNK pathway activation in the eye [71], indicating that DTRAF2 might act downstream of the TNF receptor and upstream of dTAK1 for JNK signaling. However, knockdown of either *egr* or *wgn* (the TNF/TNFR orthologs, respectively) using *UAS-RNAi* lines [64], [66] had no visible effect on Vpu-induced wing phenotypes (data not shown), suggesting that Vpu interacts with JNK signaling downstream of these components. Additionally, we found that Vpu effects in the wing might require another JNKK, dMKK4 (Figure 8), which is able to phosphorylate the JNK/BSK protein *in vitro* and activate the JNK pathway [72]. In mammals, MKK4 and MKK7 (the mammalian orthologs of *Drosophila* dMKK4 and HEP, respectively) have been reported to activate JNK synergistically [73]. In *Drosophila*, *dMkk4* has been demonstrated to act in parallel (and not redundantly) to HEP in dTAK1-mediated JNK activation in S2 cells [74]. Finally, the two JNKK, HEP and dMKK4, were shown to be phosphorylated directly by SLPR in an *in vitro* kinase assay [59]. Therefore, five regulators of JNK/BSK activation (dMKK4, HEP, SLPR, dTAK1 and DTRAF2) that have been shown in other systems to exhibit intricate relationships are also implicated in mediating the effects of Vpu. Taken together our results show that Vpu cell-autonomously activates the JNK pathway constitutively, likely *via* DTRAF2.

### IV – JNK pathway activation is the primary event triggered by Vpu to induce apoptosis and extrusion of apoptotic cells is a secondary event

Previous studies have shown that *rpr*-induced cell death was mediated by JNK activity in the *Drosophila* eye, and that *rpr* overexpression in fly S2 cultured cells led to JNK activation by promoting the degradation of DIAP1 which in turn leads to the stabilization of DTRAF1 ([54], [66]; Figure 9). Our results demonstrate that the primary event induced by Vpu to trigger apoptosis is the activation of the JNK pathway rather than DIAP1 downregulation since: (*i*) Vpu-induced *rpr* expression, DIAP1 downregulation and apoptosis all depend on JNK signaling activity, (*ii*) inhibition of caspase activity by P35 does not block Vpu-induced *rpr-lacZ* or *puc-lacZ* expression, and (*iii*) no or very little activation of the JNK pathway is observed when *diap1* function is reduced by *RNAi* or in a *diap1* heterozygous mutant background (data not shown for last point).

Vpu-induced apoptosis of epithelial cells at the A/P compartment boundary of the wing imaginal disc is associated with posterior displacement and basal extrusion of these cells, which depends on JNK/BSK function. Cell extrusion is a process that protects epithelial integrity by removing abnormal cells. *rpr*-induced cell death is correlated with basal extrusion of apoptotic cells from the wing disc epithelium [75]. In the case of wing disc cells over-expressing the *Abelson kinase* (*dAbl*) [76] or mutant for the *C-terminal Src kinase* (*dCsk*) [77], posterior cell displacement (qualified as migration of the cells) was shown to begin independently of cell death. Conversely, Moesin-depleted cells were shown to be caspase-positive while still properly integrated in the wing imaginal epithelium, and to subsequently migrate posteriorly and be excluded basally [52]. Here, similarly, Vpu-expressing cells first exhibited apoptosis since TUNEL positive cells expressing Vpu are found properly positioned within the epithelium (Figure 3g′″, arrowhead), then were displaced posteriorly and extruded basally. Importantly, in all these systems including ours, apoptosis and basal extrusion depend on JNK pathway activity. We thus propose that JNK-dependent apoptosis induced by Vpu is a primary event, whereas extrusion of apoptotic cells is a secondary effect.

### V - HIV-1, apoptosis and JNK signaling

Using the *Drosophila* wing disc as a model, we have brought to light a novel functional link between the HIV accessory protein Vpu and caspase-dependent apoptosis *via* the activation of the JNK pathway. Interestingly, the JNK pathway has also been linked to HIV-induced apoptosis in human cells. Indeed, HIV-1 infection of Jurkat cells was shown to induce the expression of MAP Kinases, including JNK, and to down-regulate the expression of anti-apoptotic factors [78]. Our work should now be pursued by testing, for example, whether JNK pathway activation detected in HIV-1 infected Jurkat cells depends of Vpu expression. JNK pathway activation should also be tested in other cell lines [21].

In the future it will be also be important to identify the target through which Vpu activates the JNK pathway in our *Drosophila* wing model. Our current data suggest that Vpu might act on DTRAF2 or upstream of DTRAF2, but do not support a role for EGR/WGN, the *Drosophila* TNF/TNFR orthologs. Therefore, it would be interesting to test a physical interaction between Vpu and dTRAF2. Establishment of a functional link between JNK and Vpu-induced apoptosis in *Drosophila* offers a new perspective for the study of Vpu effects during HIV-1 infection of human cells.

## Materials and Methods

### *Drosophila* stocks and crosses

Flies were raised on standard corn-agar medium. Except when stated in the text, flies were raised at 25°C.

*UAS-Vpu*, *UAS-Vpu-HA*, *UAS-Vpu2-6* and *UAS-Vpu2-6-HA* constructs and strains are described in [31]. Vpu2/6 is a mutant form of Vpu, in which Ser52 and Ser56 have been replaced by asparagine residues.

*Gal4* and *Lac-Z* transgenic lines used are: *en-Gal4* (BL#30564), *GMR-Gal4* (BL#9146), *1096-Gal4* (MS1096; BL#8696), *C765-Gal4* (BL#36523) *and da-Gal4* (BL#8641), *dpp-lacZ BS3.0* (BL#5527; [79]), *wg-lacZ* (BL#11205), *en-lacZ* (BL#5801), *hid-lacZ (hid^PZ 05014^*, BL#11642) and *UAS-lacZ* (BL#3955) from the Bloomington *Drosophila* stock center (Indiana) and *dpp^blnk^-Gal4* [80], *puc-lacZ* (*puc^E69^/TM3*; [55]) and *rpr-LacZ* (*rpr-11lacZ*; [81]).

To minimize the effects of the genetic background on Vpu-induced adult phenotypes, the *dpp-Gal4 UAS-Vpu/TM3 Sb* recombinant line, *UY1835* and *UAS-diap1/CyO* (a gift from B. Hay, California Institute of Technology, Pasadena, CA) transgenic lines were crossed for at least ten generations against a Canton-S reference line (the *yw^c^* strain).

Genetic interaction tests for modification of Vpu-induced wing phenotypes were performed by crossing *dpp-Gal4 UAS-Vpu/TM3 Sb* females with males of the following strains: *UAS-GFP* [82], *UY1835, UAS-diap1/CyO*, *UAS-eiger-IR* [64]; and *UAS-wengen-IR* [66] (both provided by F. Leulier), *UAS-dronc-IR* [83], *drice^17^* (strong loss-of-function; [84]) and *dronc^I24^* (amorphic allele; [85]) (both from Andreas Bergmann), *UAS-drice-IR* (7788R-1; [83]), *UAS-bsk-IR* (5680R-2; [86], [87] and *UAS-hep-IR* (4353R2; [86]), from the National Institute of Genetics stock center (NIG-Fly, Japan), *def(3L)H99/TM3Sb* (BL#1576), *UAS-p35* (BL#5072), *bsk^1^/CyO-GFP* (BL#3088), *msn^102^/TM6b* (BL#5945), *msn^06946^/TM6b* (BL#11707), *Rac1^j11^/TM6b* (BL#6674), *Rac1^j11^ Rac2^Δ^/TM6b* (BL#6677) from the Bloomington stock center and *UAS-dronc-IR* (100424), *UAS-msn-IR* (101517), *UAS-Mkk4-IR* (26928, one off-target, CG3823; [88]), *UAS-dtak1-IR* (108611), *UAS-slpr-IR* (106449), *UAS-dtraf1-IR* (21213 and 21214), *UAS-Drice-IR* (28064 and 28065) and *UAS-dtraf2-IR* (16125 and 16126; one off-target, CG14478) from the Vienna *Drosophila* RNAi Center (VDRC). *UAS-msn-IR*, *UAS-slpr-IR* and *UAS-bsk-IR* constructs were verified for their effect on dorsal closure as a control for their ability to down-regulate JNK signaling (D. Contamine, personal communication). Likewise, *UAS-dTak1-IR* and *UAS-dtraf2-IR* constructs were shown to suppress the small eye phenotype resulting from *eiger* ectopic expression in the eye with the *GMR-Gal4* driver (D. Contamine, personal communication). Other lines tested are *UAS-slimb* [89], *hep^G0107^/FM7* and *hep^r75^/FM7* (strong hypomorphic alleles, kindly provided by S. Gaumer). For each strain tested, a control cross was performed in parallel by crossing *dpp-Gal4/TM3Sb* females with males of the corresponding strain. As a control for the effect of inclusion of two UAS lines in these tests, *dpp-Gal4 UAS-Vpu/TM3 Sb* females were crossed with UAS-GFP males (Figure S1).

The effect of the downregulation of *slimb* was assayed by crossing *UAS-slimb-IR* males (107825 from the VDRC and *UAS*-3412R1 from NIG Fly) with *dpp-Gal4/TM3Sb* females. The same procedure was applied to test downregulation of *thread/diap1* (with *UAS-diap1-IR*, [83]) and *reaper* (*UAS-rpr* provided by S. Gaumer).

For *lacZ* staining, immunostaining and TUNEL labeling of imaginal discs, *dpp-gal4 UAS-Vpu/TM6TbSb*, *UAS-LacZ*; *dpp-Gal4/TM6TbSb*, *enGal4/CyO*; *puc-lacZ/TM6TbSb*, *enGal4*; *rpr-lacZ*, *UAS-Vpu UAS-bsk-IR/TM6TbSb*, *bsk^1^/CyoGFP*; *UAS-VPU/TM6TbSb*, *UAS-dronc-IR/CyoGFP*; *UAS-VPU/TM6TbSb*, *UAS-VPU UAS-hep-IR/TM6TbSb*, *UAS-p35*; *dpp-Gal4 UAS-Vpu/TM6TbSb*, *UAS-p35*; *rpr-lacZ*, *UAS-p35*; *puc-lacZ/TM6TbSb*, *hep^G0107^/FM7-GFP*; *dppGal4 UAS-Vpu/TM6TbSb*, *MS1096*; *UAS-Vpu/TM6TbSb* and *hep^r75^/FM7-GFP*; *UAS-Vpu* recombinant lines were used.

### X-Gal-staining, immunostaining and microscopy

β-Galactosidase assays and immunofluorescence staining of third instar larval imaginal discs were carried-out using standard protocols. The following primary antibodies were used: mouse anti-Diap1 (1∶100 ) (kind gift from B. Hay), mouse anti-β-Galactosidase (1∶200) (Developmental Studies Hybridoma Bank), rabbit anti-β-Galactosidase (1∶500) (Rockland, Immunochemicals, Inc), rabbit anti-Vpu (1∶50) (produced against a peptide located in the C-terminus of the protein and kindly provided by K. Levesque) and rabbit anti-ACTIVE JNK (1∶50) (Promega). When using this last antibody, larvae were dissected in phosphate buffer on ice and directly transferred to fixation buffer on ice during a maximum of 10 minutes before standard fixation procedure.

Fluorescently labeled Alexa 488, 568 and 647 secondary antibodies (1∶500) were used (Molecular Probes, Invitrogen). Atto647N-Phalloïdin (Fluka) was used at 1∶200 for 30 minutes to label the F-actin network. Discs were mounted in Fluorescence Mounting Medium (Dako).

Discs stained for β-galactosidase activity were photographed on a LEICA MRD microscope with standard Nomarski optics. For immunostaining and TUNEL labeling, images were captured using a NIKON TE2000-U inverted confocal microscope, processed and treated with ImageJ64 and Adobe Photoshop CS2 software, using identical settings for all samples of the same experimental series. Transverse sections were computationally generated after reslicing the confocal stacks using the ImageJ64 reslice tool.

### TUNEL assay

*dpp-Gal4 UAS-Vpu/TM6TbSb* females were crossed either with *yw^c^*, *UAS-p35*, *puc-lacZ/TM6TbSb or UAS-bsk-IR/TM6TbSb* males. *dpp-Gal4/TM6TbSb* females were crossed with the same males as a control. *dpp-Gal4/TM6TbSb* females were crossed with *UAS-Vpu2-6* males and with *yw^c^* males as a control. Apoptotic cells were detected using the ApopTag® Red In Situ Apoptosis Detection Kit (Chemicon International). TUNEL staining (which labels DNA breaks) was performed following manufacturer's instructions. In the same experiment, immunodetection of either β-galactosidase from the *puc^E69^* construct or Vpu was carried out.

### Acridine Orange staining of wing discs

For Acridine Orange staining (which reveals nuclear chromatin clumps), third instar larvae were stained for 2 min in 100 ng ml^−1^ Acridine Orange (Molecular Probes). Mounted samples were observed immediately by fluorescence microscopy in the green channel.

### Statistical analyses of adult wing phenotypes

We used a Chi-square test to determine whether a mutant background or *RNAi*-mediated extinction of a candidate gene statistically modifies the distribution of adult wing phenotypes resulting from Vpu expression driven by *dpp-Gal4*. The null hypothesis is that the probability of having the same distribution among the four phenotypical classes (strong, medium, weak and very weak; Figure 3A) is the same for the two genotypes compared. Three different controls (*yw^c^*, *w^1118^* and 60100VDRC lines) were used to evaluate the effect of the genetic background on Vpu-induced phenotypes. This analysis led us to choose a threshold of p<10^−4^ for significance in the test of comparison between genotypes. This high level of stringency allowed us to circumvent the effects of genetic background. *yw^c^*, *w^1118^* and *V60100* (VDRC) fly strains were used as controls when appropriate. At least two independent experimental series were carried out for each mutant line tested. Similar results were observed for females and males in the progeny of each cross (only the results for females are shown in the graphs).

### Two hybrid assay

Plasmids: a DNA fragment encoding the WD1 region of SLIMB (amino acids (aa) 192 to 242) was cloned by PCR between the *EcoRI* and *XhoI* sites of pJG4-5. The region encoding the cytoplasmic domain (aa 28 to 81) of Vpu or of the Vpu*2-6* mutant were excised from pGBT10 vectors [10] and were cloned between the *EcoRI* and *XhoI* sites of pEG202. The pJG4-5-derived plasmids were introduced into the *RFY206 Saccharomyces cerevisiae* strain while the vectors derived from pEG202 were introduced into the *EGY48* strain already transformed with the plasmid pSH18-34 (which carries the *lacZ* reporter gene). The method used for the two hybrid assay was performed as in [90]. All PCR constructs were sequenced.

### Western blot and co-immunoprecipitation assay

Ten third instar larvae were lysed with a Dounce homogenizer in cold lysis buffer (50 mM Tris-HCl PH 7,4, 150 mM NaCl, 0,5% NP40, 1 mM DTT). The lysate was then centrifugated 5 min at 18000 rpm. To prepare total extracts, the supernatant was then incubated with 10% TCA for 10 min at 4°C. After centrifugation at 18000 rpm, the precipitated proteins were resuspended in SDS-sample buffer. For co-immunoprecipitation assays, 100 µl of the supernatant were then collected and incubated overnight at 4°C with rat anti-SLIMB (1∶100) [89]. Complexes were immunoprecipitated using protein G sepharose (Immunoprecipitation Starter Pack, Amersham, Biosciences). Bound proteins were eluted with SDS-sample buffer. Proteins were then separated by 15% denaturing SDS/PAGE and analyzed by immunoblotting using an anti-HA antibody (rat anti-HA peroxydase, High affinity 3F10, Roche; 1/1000). Primary antibody was detected with an anti-rat horseradish peroxidase-conjugated (Jackson ImmunoResearch) revealed by enhanced chemiluminescence (ECL; Amersham).

To quantify Vpu and Vpu2-6 expression levels, 20 wing imaginal discs were lysed, centrifugated and incubated with Laemmli buffer, DTT 0,01 M. 15 µl of pure extract or dilutions (1/2 and 1/5) were then separated on a 15% denaturing SDS/PAGE and analyzed by immunoblotting using rabbit anti-Vpu (1∶1000) (kindly provided by K. Janvier) and detected with an anti-rabbit horseradish peroxidase-conjugated secondary antibody (Bethyl; 1/12000). Vpu and Vpu2-6 proteins were quantified using Integrated Density method in ImageJ64 software.

### Genetic screen to isolate modifiers of Vpu eye- and wing-phenotypes

We carried out a gain-of-function (GOF) screen for genes whose deregulation causes alterations in Vpu-induced adult wing and eye phenotypes. The mutagen used was a *P*-element vector, *P[Mae-UAS.6.11]*, carrying a *yellow* ^+^ gene as a transformation marker and GAL4 binding sites (UAS) at the 5′ end (called *P[yellow^+^-UAS]* in the text), oriented towards adjacent genomic sequences (Merriam J. 1997. FlyBase). We participated in the production of a collection of *Drosophila P[Mae-UAS.6.11]* insertion lines named here *UYi*, where *i* is the number of the line [91]. The GOF screen was performed by crossing *dpp-Gal4 UAS-Vpu* or *GMR-Gal4; UAS-Vpu* isogenized females with males from a *UYi* line. Control crosses (*dpp-Gal4 or GMR-Gal4* females crossed with males from the same *UYi* lines) were performed in parallel. To characterize the modifier genes, flanking genomic DNA (at the 5′ end of the *P* element) was isolated from positive UYi lines by inverse PCR (according to the procedure described in the BDGP resources at www.fruitfly.org/about/methods/inverse.pcr.html) and sequenced. Sequences were analyzed using the BLASTN program. The molecular characterization the *UY1835* line showed that the *P* element is inserted in the 5′ UTR sequence of the *thread*/*diap1* gene (+2088 bp from the transcription start site of the B transcript), in the correct orientation to allow the expression of the encoded DIAP1. We confirmed that this insertion allowed rescue of cell death resulting from overexpression of the pro-apoptotic gene *reaper* (*rpr*) in the *Drosophila* eye (data not shown) as previously shown with the overexpression of a *UAS-diap1* construct [64].

## Supporting Information

Figure S1 **Vpu-induced wing phenotypes are not suppressed by an additional UAS transgene.** The distributions of wing phenotypes are not statistically different (Chi^2^ test) in the progeny of a UAS-Vpu X UAS-GFP cross (red; n = 337) from that of a UAS-Vpu X yw^c^ control cross (yellow; n = 195): p = 0.21, showing that the inclusion of an additional *UAS* transgene is not sufficient to suppress Vpu-induced wing phenotypes.
(TIF)

Figure S2 **Caspase activity is not required for Vpu-induced** ***rpr*** **expression or JNK pathway activation.** Expression of *rpr-lacZ* (A and B) and *puc-lacZ* (C and D) revealed by X-Gal staining in *dpp-Gal4 UAS-Vpu/rpr-lacZ* (A), *UAS-p35/+*; *dpp-Gal4 UAS-Vpu/rpr-lacZ* (B), *dpp-Gal4 UAS-Vpu/puc-lacZ* (C) and *UAS-p35/+; dpp-Gal4 UAS-Vpu/puc-lacZ* (D) wing imaginal discs. The expression domains of *rpr*- and *puc-lacZ* reporters are expanded when *p35* is co-expressed with Vpu (B and D compare to A and C, respectively), suggesting that *p35* allows survival of Vpu-expressing cells in which *rpr* and *puc* promoters have been activated.
(TIF)

Table S1 **Molecular analysis of some GOF modifiers of the Vpu-induced wing and eye phenotypes.**
(PDF)
