# Supplementary figures and images for: The HIV-1 Vpu Protein Induces Apoptosis in Drosophila via Activation of JNK Signaling
Source: PLoS One. 2012 Mar 29;7(3):e34310. doi: 10.1371/journal.pone.0034310 (PMC3315533; doi:10.1371/journal.pone.0034310)

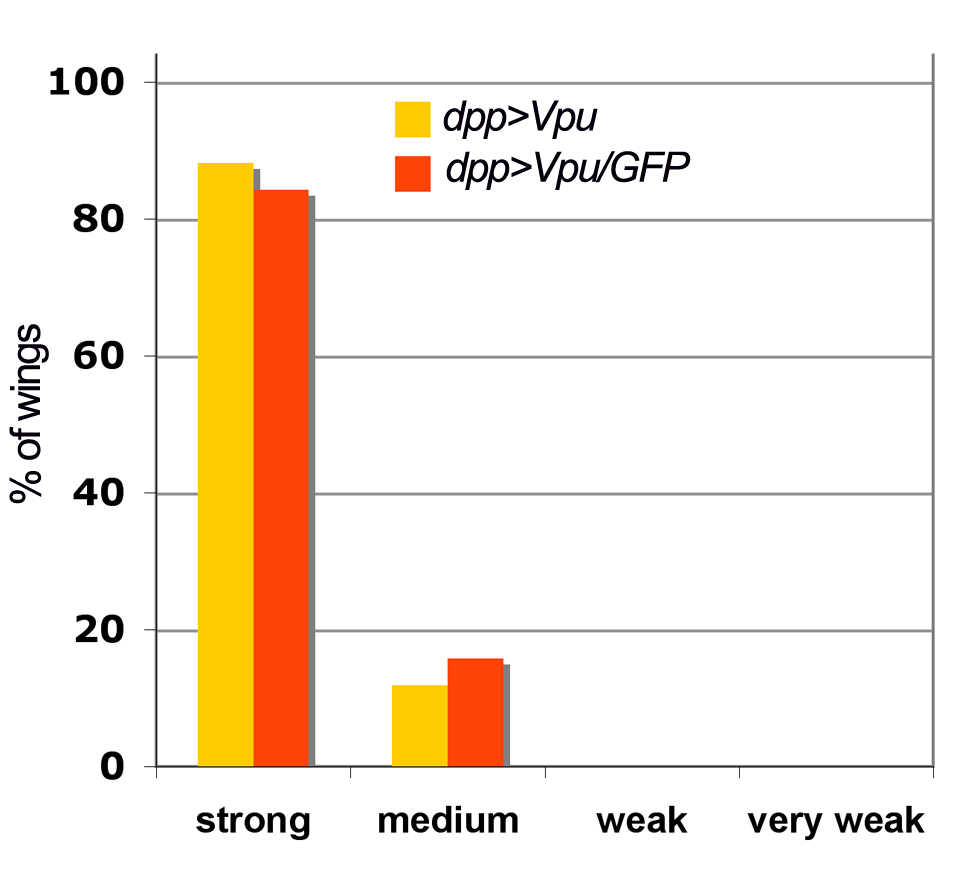

Supplement: Figure S1 — Vpu-induced wing phenotypes are not suppressed by an additional UAS transgene. The distributions of wing phenotypes are not statistically different (Chi2 test) in the progeny of a UAS-Vpu X UAS-GFP cross (red; n = 337) from that of a UAS-Vpu X ywc control cross (yellow; n = 195): p = 0.21, showing that the inclusion of an additional UAS transgene is not sufficient to suppress Vpu-induced wing phenotypes. (TIF) [file pone.0034310.s001.tif]

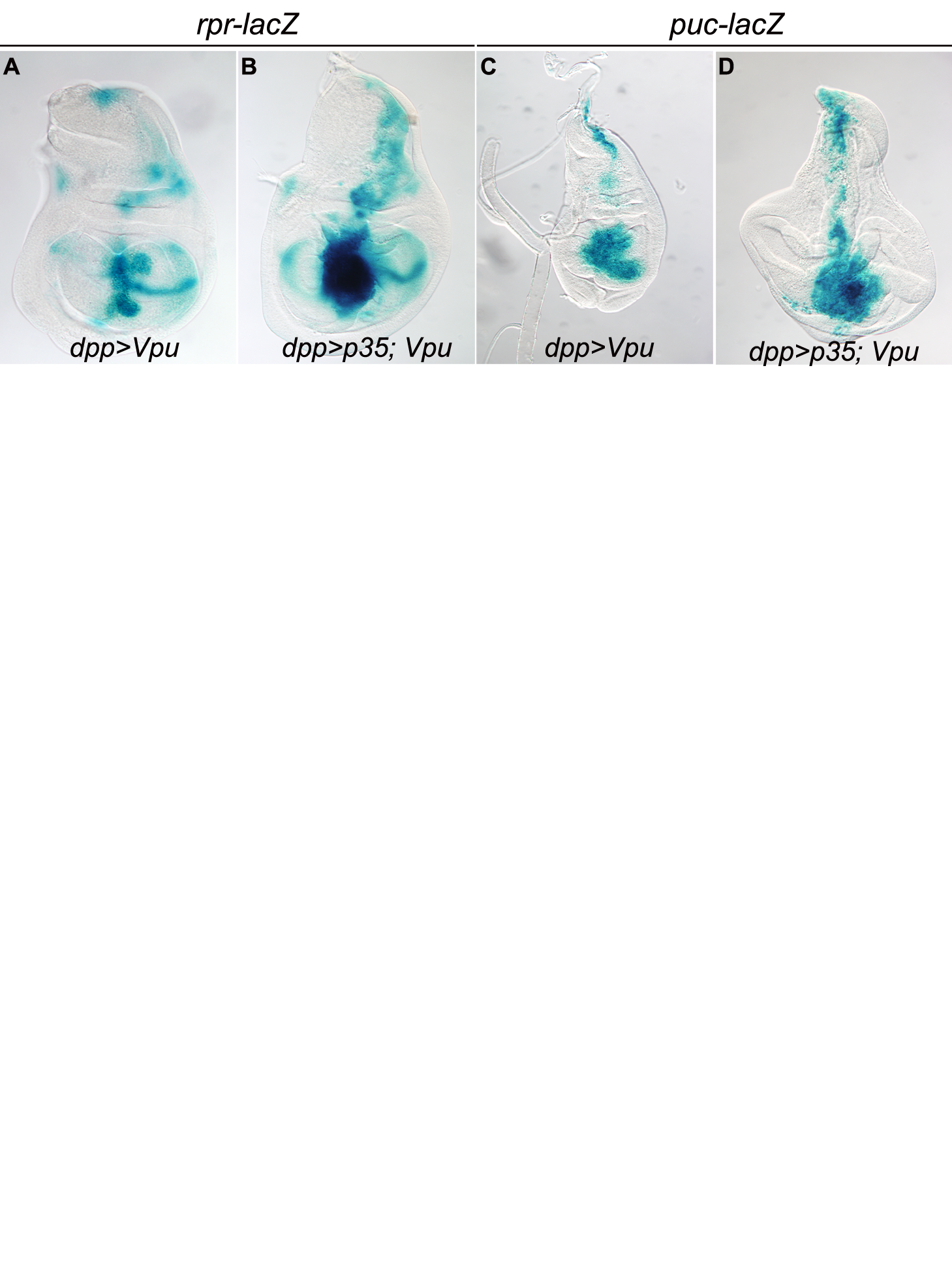

Supplement: Figure S2 — Caspase activity is not required for Vpu-induced rpr expression or JNK pathway activation. Expression of rpr-lacZ (A and B) and puc-lacZ (C and D) revealed by X-Gal staining in dpp-Gal4 UAS-Vpu/rpr-lacZ (A), UAS-p35/+; dpp-Gal4 UAS-Vpu/rpr-lacZ (B), dpp-Gal4 UAS-Vpu/puc-lacZ (C) and UAS-p35/+; dpp-Gal4 UAS-Vpu/puc-lacZ (D) wing imaginal discs. The expression domains of rpr- and puc-lacZ reporters are expanded when p35 is co-expressed with Vpu (B and D compare to A and C, respectively), suggesting that p35 allows survival of Vpu-expressing cells in which rpr and puc promoters have been activated. (TIF) [file pone.0034310.s002.tif]
