# Supplementary material for: The HIV-1 Vpu Protein Induces Apoptosis in Drosophila via Activation of JNK Signaling
Source: PLoS One. 2012 Mar 29;7(3):e34310. doi: 10.1371/journal.pone.0034310 (PMC3315533; doi:10.1371/journal.pone.0034310)
Supplement: Table S1 — Molecular analysis of some GOF modifiers of the Vpu-induced wing and eye phenotypes. (PDF) [file pone.0034310.s003.pdf]

**TABLE S1. Molecular analysis of some GOF modifiers of the Vpu-induced wing and eye phenotypes**

| Uyi[1] | Gene[2] | Insertion site[3] | Molecular information / Function | wing[4] | eye[4] | Human homolog[5] |
|---|---|---|---|---|---|---|
| **Class 1: Transcriptional factors** | | | | | | |
| 146 | *cropped (crop)* | 2nd intron | Unknown domain / Function | S | 0 | *TFAP4* |
| 181 | ***tramtrack (ttk)*** | -11700 pb | Transcription factor / Peripheral nervous system | S | 0 | BTB/POZ family proteins |
| 195 | *lola* | According to transcripts | Transcription factor / Axonal guidance | S | 0 | BTB/POZ family proteins |
| 1238* | *su(H)* | +32pb (AS) | Transcription activator / Notch transduction | E | E | *CBF1/RBPJ* |
| 1526 | *mes-4* | +40pb (AS) | Histone methyl transferase / Gene silencing | S | 0 | *NSD1* |
| 1877 | *emc* | +208pb | Transcription repressor / Cell-cell signaling; cell proliferation | L | 0 | *Inhibitor of DNA binding4* |
| 1170 | *shnurri (shn)* | -3490pb (AS) | Transcription factor / Wing morphogenesis; Cell proliferation | E | 0 | *HIV-EP3* |
| **Class 2: Translation, splicing** | | | | | | |
| 1028 | *Rpl7* | -1983pb | Structural constituent of ribosome / Mitosis | 0 | S | *RPL7* |
| 1276** | *eIF3-S10* | -85pb | Translation initiation factor / Mitosis | S | E | *EiF3A* |
| 1652* | *heph* | 4th intron | Translation repressor / Notch signaling pathway | E | E | *hnRNP1/PTB1* |
| 3006 | ***fusilli*** | (AS) | mRNA binding / EGFR signaling | S | 0 | *ESRP1* |
| 1051** | *waharan* | -38bp (AS) | Aminoacyl-tRNA ligase activity; ATP binding / Multiple processes | E | S | - |
| **Class 3: Apoptosis** | | | | | | |
| 1206 | *mir-278 stem loop* | NK | miRNA-gene / Negative regulation of apoptosis | S | 0 | - |
| 15 | *anterior open (aop)* | -107bp | Transcription repressor / Positive regulation of apoptosis | S | L | *ETV6* |
| 1835 | ***thread (th)/diap1*** | +2088bp | Apoptosis inhibitor / Apoptosis | S | 0 | *IAP* |
| 1027* | *malic enzyme (men)* | 2nd exon | Malate dehydrogenase / Regulation of apoptosis | S | S | *malic enzyme I cytosolic* |
| 1238* | *l(2)35Bg* | -713bp | Unknown / Apoptosis inhibitor related | E | E | *Ciapin1* |

| | | | | | | |
|---|---|---|---|---|---|---|
| **Class 4: Ubiquitin, Proteasome** | | | | | | |
| 1339* | *partner of paired (ppa)* | -45bp | Unknown domain / Ubiquitin-dependent protein catabolic process | E | E | *FBXL14* |
| 1323 | *CG2218* | -186bp | Ubiquitin-protein ligase activity / Ubiquitylation | S | 0 | *UBOX5* |
| **Class 5: Signaling pathways** | | | | | | |
| 1261 | *CG14945* | -45bp | Phospholipase C activity / intracellular signal transduction | 0 | L | *Phosopholipase C family* |
| 1726 | *target of wingless(tow)* | +126bp (AS) | Unknown domain / Regulation of Smoothened signaling pathway | S | 0 | - |
| 1755 | *pxb* | -43bp (AS) | Unknown domain / Smoothened signaling pathway | S | 0 | - |
| 1033 | *eathbound 1 (ebd1)* | 1st exon | DNAbinding / Positive regulation of WG signaling | 0 | S | - |
| **Class 6: Metabolism** | | | | | | |
| 1283 | *CG2098* | -364bp | Ferrochelatase / Protoporphyrinogen IX biosynthetic process | 0 | E | *FECH* |
| 1030 | *CG7910* | +162bp (AS) | Fatty acid amide hydrolase / Unknown function | S | 0 | *fatty acid amide hydrolase* |
| 1730 | *O-GlcNAcase (oga)* | +54bp (AS) | Hyaluronoglucosaminidase / Unknown function | 0 | L | *MGEA5* |
| 1751 | *Ssadh* | +2281bp (AS) | Succinate-semialdehyde dehydrogenase / Gamma-aminobutyric acid catabolic process | S | 0 | *ALDH5A1* |
| **Class 7: Cell adhesion, extracellular matrix, cytoskeleton** | | | | | | |
| 1228** | *CG14394* | -2682bp | Unknown domain / Tissue regeneration; Cell adhesion | S | E | *Ninjurin 2* |
| 1526 | *CG5514* | -800bp | Structural constituent of cell wall / Unknown function | S | 0 | - |
| 1702 | *Kuzbanian (Kuz)* | 1st intron | Metallo-endopeptidase / Peripheral nervous system | S | 0 | *ADAM10* |
| 1332 | ***scr64*** | +1500bp | Tyrosine kinase / Actin filament-based process | 0 | E | *FYN* |
| 1116 | *viking (vkg)* | +8bp | Extracellular matrix structural constituent / Skeletal muscle development | 0 | L | *Type IV collagen* |
| 1290 | *Laminine B1 (LanB1)* | -39bp | Unknown / Organ development; Cell migration; Basement membrane assembly | 0 | S | *Laminin B* |
| **Class 8: Traffic** | | | | | | |
| 3113* | ***liprin alpha*** | -48bp | Protein binding / Axon guidance | L | E | *PPFIA1/Liprin* |

| | | | | | | |
|---|---|---|---|---|---|---|
| 1051** | *c(3)G* | -410bp | Reciprocal meiotic recombination | E | S | *Restin/CLIP-170* |
| 1228 | *Past1* | +562bp | Calcium ion binding / Endocytosis | S | 0 | *HPAST1/EHD1* |
| 1824 | *CG8451* | -374bp | Multivitamin transmembrane transporter / Membrane transport | S | 0 | *SSSF* |
| **Class 9: Others** | | | | | | |
| 1199 | ***grapes (grp)*** | -99bp | Ser/thr kinase / Cell cycle; DNA damage checkpoint. | 0 | E | *CHEK1* |
| 1213 | ***grapes (grp)*** | -183bp | Ser/thr kinase / Cell cycle; DNA damage checkpoint. | 0 | E | *CHEK1* |
| 1776 | ***grapes (grp)*** | -200bp | Ser/thr kinase / Cell cycle; DNA damage checkpoint. | 0 | E | *CHEK1* |
| 1324 | *CG4552* | +634bp (AS) | Rab GTPase activator activity / Phagocytosis, engulfment | S | 0 | *TBC1D23* |
| 1751 | *CG4673* | -900bp (AS) | Structural constituent of nuclear pore / Neurogenesis | S | 0 | *NPLOC4* |
| 5377 | *amnesiac (amn)* | +1010bp | Neuropeptide hormone / Learning, memory;Antimicrobial humoral response | E | 0 | - |
| 1702 | *B4* | -1843bp (AS) | Unknown domain / Imaginal disc development; Circadian rythm. | S | 0 | - |
| 3110 | *cht11* | +1269bp | ChitinaseII / Chitin catabolic process | E | 0 | - |
| 1348 | *CG8003* | -448bp | Zinc ion binding / Unknown function | E | 0 | *ANKMY2* |
| 1730 | *CG3353* | -100bp | Zinc ion binding / Unknown function | 0 | L | *SMYD5* |
| 1001 | *CG32369* | 1st intron (AS) | Zinc ion binding / Proteolysis | S | 0 | *LONRF2* |
| 1657 | *Esyt2* | -8553bp | C2 membrane targeting protein / Unknown function | 0 | L | *ESYT2* |

[1] P[yellow+ -UAS] insertion line number

[2] In **bold** are the genes verified to be deregulated by the insertion of the P[yellow$^{+}$ -UAS] element

[3] Insertion site corresponds to the location of the P element relative to the known/putative transcriptional start site of the candidate gene. All P element insertions are in the sense transcriptional orientation relative to the candidate gene unless specified by (AS). NK: transcription start site not known

[4] Modification of the Vpu-induced phenotype in the wing or in the eye. S: suppressor, E: enhancer, L: léthal, 0: no modification.

[5] - No human homolog identified

* Insertion lines that modified both wing and eye Vpu-induced phenotypes in the same direction

** Insertion lines that modified wing and eye Vpu-induced phenotypes in the opposite direction
